# A novel MCDM approach for design concept evaluation based on interval-valued picture fuzzy sets

**DOI:** 10.1371/journal.pone.0294596

**Published:** 2023-11-27

**Authors:** Qing Ma, Hongyuan Sun, Zhe Chen, Yuhang Tan

**Affiliations:** Shandong Jiaotong University, Jinan, China; Istanbul University: Istanbul Universitesi, TURKEY

## Abstract

The assessment of design concepts presents an efficient and effective strategy for businesses to strengthen their competitive edge and introduce market-worthy products. The widely accepted viewpoint acknowledges this as a intricate multi-criteria decision-making (MCDM) approach, involving a multitude of evaluative criteria and a significant amount of data that is frequently ambiguously defined and subjectively influenced. In order to tackle the problems of uncertainty and fuzziness in design concept evaluation, our research creatively combines interval-valued picture fuzzy set (IVPFS) with an MCDM process of design concept evaluation. Firstly, this study draws on the existing relevant literature and the experience of decision makers to identify some important criteria and corresponding sub-criteria and form a scientific evaluation indicator system. We then introduce the essential operational concepts of interval-valued picture fuzzy numbers (IVPFNs) and the interval-valued picture fuzzy ordered weighted interactive averaging (IVPFOWIA) operator. Thirdly, an entropy weighting method based on IVPFS is proposed in this research to calculate the weights of criteria and sub-criteria, and based on this, an integrated IVPF decision matrix is further constructed based on the presented IVPFOWIA operator. Finally, the best design concept alternative is selected by applying the extended TOPSIS (Technique for Order of Preference by Similarity to Ideal Solution) approach with IVPFS. The IVPFS combined with improved MCDM method have been proven to be superior in complex and uncertain decision-making situations through experiments and comparative assessments. The information ambiguity in the evaluation of design concept is well characterized by our augmentation based on IVPFS.

## 1. Introduction

Design concept is the initial stage of a product lifecycle. Although this process is normally very short in the long lifecycle of a product, about 80% of sustainability and other impacts are decided in this stage. Because decisions and requirements are made in this stage, design concept evaluation is one of the most critical steps in the product design stage. Once the design concept is selected, most impacts such as novelty, feasibility and sustainability, as well as maintenance and disposal methods, are determined [1]. Hence, design concept evaluation is a complicated process, the complexity of product concept design evaluation aries from considering multiple factors, dealing with uncertainty, making decisions based on variable criteria, and collaborating with diverse teams, often assigned by decision support tools [2]. Problems like design concept evaluation are commonly defined as an MCDM problem.

As an important research field of MCDM [3], effective related approaches have already been developed and applied for design concept evaluation [4, 5]. Some studies are based on linguistic-based information, in which experts give their preferences based on a multiple-scale index [6]. However, as the raw data are usually imprecise, incomplete or subjective [4], methods based on fuzzy sets are integrated for design concept evaluation. Rough set is an important MCDM method in design concept evaluation to transform crisp information into rough intervals, and select the optimal alternative by the decision matrix based on rough numbers [7]. Rough-TOPSIS [8, 9], Rough-VIKOR [10] and Rough-AHP and Rough-TOPSIS [11] are then applied in design concept evaluation. Other MCDM methods such as fuzzy method [12, 13], grey theory [14] and vague set [15] based on linguistic information are also implemented in specific decision-making of design schemes. However, as the experts are not always familiar with all the attributes of a product, it is very hard for experts to give an exact preference for a multiple-scale. Membership methods provide another way of dealing with MCDM problems [16]. Intuitionistic fuzzy set (IFS), as a widely applied vote-based approach, has shown its advantage for design concept evaluation [17, 18]. Experts make their decisions using agree or disagree for each attribute and each alternative. Even though the IFS has achieved ideal results, it is not sufficient to cover all the situations of experts’ decisions with only two options of agree or disagree. When an expert feels unfamiliar with an attribute, the expert has the authority to abstain or “sit on the fence”. Once these circumstances are considered, the decision information environment becomes more vague.

In order to address the issues of fuzziness and uncertainty in design concept evaluation, this study combines IVPFNs with improved TOPSIS to present a systematic framework for decision-making. The purpose of this research is as follows: (1) to present IVPFNs to handle ambiguity and subjectivity in decision-making, and enhance the objectivity of evaluation outcomes. (2) to put forward improved TOPSIS method based IVPFNs to strengthen the MCDM method and to underline the superiority of IVPFNs.The main contributions of our manuscript include:

1. We have successfully extracted and described the fuzziness and uncertainty in the design concept evaluation process based on the IVPFS theory. More precisely, this work offers a practical technique for transforming qualitative assessment data input into IVPFNs for design idea evaluation, enabling adaptable decision-making processes.
2. Our research develops and applies a new aggregation operator to integrate IVPFS, taking advantage of OWIA to eliminate the correlation of indicators. As is well known, no literature has examined the use of the OWIA operator and IVPFS together while evaluating design concepts.
3. We have integrated improved TOPSIS method with IVPFS theory to evaluate some design concept schemes to determine the best one. This algorithm addresses the limitation of traditional TOPSIS that cannot reflect the dynamic changes in the evaluation index sequence, ensures the accuracy of decision results.

This is how the study’s reminder is put together: Section 2 reviews the literature. Section 3 sets out several key IVPFS concepts, clarifies some fundamental and typical IVPFN operating laws, and creates the IVPFOWIA operator. A unique framework for evaluating and choosing design concept alternatives is proposed in Section 4 based on the improved TOPSIS method with IVPFS. Section 5 provides a case study to demonstrate the scientific validity as well as the feasibility of the algorithms proposed in this study. Section 6 outlines a summary of the full manuscript and predictions for potential future applications.

## 2. Literature review

Based on the IVPFS, IVPFOWIA and the extended TOPSIS method, the purpose of our research is to evaluate design concept schemes. Therefore, the literature review has three sections: (1) research on IVPFS, (2) research on IVPFOWIA, and (3) research on ranking the design concept alternatives through an extended TOPSIS method under IVPFS.

### 2.1. Interval-valued picture fuzzy set

With the advent of the information age and the rapid development of computer technology, huge and complex fuzzy datasets have appeared in many fields of daily life [19]. To address practical uncertainties, numerous scholars have formulated classical theories that align with real-world requirements. For example, since the emergence of fuzzy sets (FS), it has quickly become a hot spot in the study of uncertainty theory, and has generated research and discussion by scholars [20]. To facilitate specific applications, various extended forms of fuzzy sets have been put forth. For example, Yager first proposed theory of Pythagorean fuzzy set to describe the uncertainty issues of experts in MCDM. Subsequently, numerous scholars conducted research on the Pythagorean fuzzy set theory. A responsible enterprise not only focuses on its own development, but also on the protection of the ecological environment. Green supplier selection contributes positively to environmental protection. Therefore, Mostafa et al.[21] conducts research on Python fuzzy sets, they apply Pythagorean fuzzy set and TOPSIS method to determined the best supplier. Morsy [22] proposed a method that incorporates Pythagorean Fuzzy Number into risk return rate, portfolio risk amount, and expected return rate. Finally, an example was used to verify the effectiveness of the proposed method. In 1986, Atanassov [23] first proposed the theory of IFS, which mainly solves the indecision phenomenon in which experts may not know enough about the decision object or other factors in the decision-making process. IFS has three parts: a membership degree, a non-membership degree and an uncertainty degree. Because of its neutrality, IFS is more widely used in daily decision-making than fuzzy set theory. As the decision-making object becomes more complex, IFS theory cannot be solved. Taking expert voting as an example, there may be four possible outcomes: support, neutrality, opposition, and abstention. Therefore, in 2013 Cuong et al.[24] proposed an extended and new fuzzy set-PFS to deal with the above situation.

However, in MCDM problems, due to the limitations of experts’ understanding of decision-making objects and the ambiguity of the decision-making environment, experts can give an interval number rather than a specific real number when making a decision. Therefore, in 2013, Cuong [24] proposed IVPFS theory, so that membership, neutrality, non-membership and abstention can be represented by interval numbers to enhance the credibility of decision-making results. In certain real-world applications, IVPFS theory, an extension of PFS, are more adept at handling and modeling inconsistent, indeterminate, and incomplete data [25–27]. Therefore, it becomes very important to study the group decision problem under the IVPFS.

### 2.2 Interval-valued picture fuzzy ordered weighted interactive averaging operator (IVPFOWIA)

The aggregation operator is a particularly efficient technique for solving MCDM problems. To clarify concerns with decision-making, many scholars have studied aggregation operators in recent years. For example, Wei [28] put forward some aggregation operators under picture fuzzy set, then used these operators to deal with MCDM problems. In final, a typical example was used to demonstrate the effectiveness of the proposed operators. Due to the advantages of Archimedes operator and Dombey operator in providing appropriate generalization ability and flexibility in aggregating information, respectively. Saha et al. [29] concentrate on the use of these operators in the classifier’s aggregate similarity calculation. Finally, by comparing with previous operators, it is verified that the proposed operator has higher accuracy. Senapati et al. [30] introduced the Aczel-Alsina (AA) weighted geometric operator and the AA ordered weighted geometric operator for IFS, the effectiveness of the proposed operator was verified through two typical cases. Liu et al.[31] first introduced the Archimedean Heronian (AH) operator and the weighted Archimedean Heronian (WAH) operator for IFS. Then, they put in multiple attribute group decision making approach based on these operators. Similarly, verify the effectiveness of the proposed operator through its application in typical cases. Due to the stability and flexibility advantages of the Frank aggregation operator, Zeng et al. [32] proposed a Frank operator for the q-rung orthopair fuzzy set. Besides, they discuss the some properties,such as idempotency, commutativity, monotonicity and boundedness. Batool et al. [33] proposed a novel decision-making approach based on aggregation operators under Pythagorean probabilistic hesitant fuzzy number. In addition, they also conducted a detailed discussion on some properties of the proposed operator. Regarding the issue of defects in some operators, Luo and Long [34] proposed a weighted geometric operator and an ordered weighted geometric operator for a picture fuzzy set (PFS). Finally, they use the aggregation operators to deal with MCDM problems. Garg [35] put forward various fuzzy picture aggregation operations, and combined them with classic methods to solve practical problems. Nevertheless, the aforementioned aggregate operator merely takes the attributes’ independence into account; in reality, many attributes will be connected in varying degrees like complementary relationships, redundancy, preference relationships, etc. In our study, we applied the OWIA operator to IVPFS, taking advantage of OWIA to eliminate the correlation of indicators in the process of MCDM.

### 2.3. Improved TOPSIS method

MCDM is a decision-making approach used to evaluate and select the best option from a set of alternative solutions when multiple criteria or factors need to be considered [36]. MCDM methods help decision-makers rank and prioritize options based on these criteria, making it easier to make informed and objective decisions [37–40]. This approach is widely applied in various fields such as engineering, management, environmental planning, and medical decision-making. Through MCDM, decision-makers can better balance various factors to make wiser decisions.

MCDM methods include the weighted summation method, weighted product method, TOPSIS method [41], the analytical hierarchy process (AHP) [42], the grey relation projection method (GRP) method [43], utility theory, graphical tools, quality function deployment (QFD) and fuzzy logic method [44]. In 1981, the TOPSIS technique was initially introduced as a prevalent method for MCDM. TOPSIS method is used to select the best choice among a set of schemes. The basic idea of the TOPSIS method is to assume positive and negative ideal solutions, calculate the distance between each sample and the positive and negative ideal solutions, obtain their relative closeness to the ideal solution (i.e., the closer they are to the positive ideal solution, the farther they are to the negative ideal solution), sort each solution, and ultimately determine the optimal solution. TOPSIS is widely applied in engineering, management, and decision sciences to effectively address multi-criteria decision problems. However, TOPSIS method only considers the Euclidean distance between indicators, and cannot reflect the dynamic changes of evaluation index series. There are shortcomings in decision analysis, especially for dynamic series. Grey correlation analysis can reflect the internal changes of each scheme, making up for the shortcomings of the TOPSIS method. In our research, the TOPSIS model is combined with the grey correlation method to improve TOPSIS, defining a new set of positive and negative ideal points and a formula for relative closeness. The improved TOPSIS model solves the problem of large fluctuations in data by calculating the degree of correlation between evaluation objects. When calculating Euclidean distance, it avoids the phenomenon of disordered equidistant sorting between sample points and positive and negative ideal points, and has strong stability and objectivity.

## 3. Basic preliminaries

In this section, we go through a number of fundamental concepts to give the necessary background information.

### 3.1. Picture fuzzy set

As an important extension of IFS, PFS has received increasing academic attention in recent years. Compared to FS [20] and IFS, PFS is more flexible and practical for expressing and processing fuzzy information. PFS takes into account the support, uncertainty, opposition and abstention of decision makers when evaluating alternatives in real-life scenarios, and expresses the ambiguity and uncertainty of decision makers about the decision problem they face in terms of four aspects: positive membership degree, neutral membership degree, negative membership degree and refusal membership degree [24].

The following are significant definitions and related properties of PFS.

**Definition 1** [24] Let *X* be a non-empty set, then it is said that:

$$A=\{\langle x,\mu_{A}(x),\eta_{A}(x),v_{A}(x)\rangle |x\in X\}$$ (1)

Where the terms *μ*_*A*_: *A*, η_A_: A, *v*_*A*_: *A*∈[0,1] denote the degrees of positive, neutral, and negative membership of an element *x* in *X*, respectively. Additionally, *μ*_*A*_, η_A_ and *v*_*A*_ meet the following requirement:

$$0\leq \mu_{A}(x)+\eta_{A}(x)+v_{A}(x)\leq 1$$ (2)

And $\xi_{A}(x)=1-(\mu_{A}(x)+\eta_{A}(x)+v_{A}(x))$ is said to be refusal membership degree of an element *x* in *X*. If ξ_A_(x) = 0, in this case *A* returns to the IFS.

**Definition 2** [24] Suppose that θ_1_ = (μ_1_, η_1_, v_1_) and θ_2_ = (μ_2_, η_2_, v_2_) are two different PFNs, then

1. $\theta_{1}⊕\theta_{2}=(\mu_{1}+\mu_{2}-\mu_{1}\mu_{2},\eta_{1}\eta_{2},v_{1}v_{2})$;
2. $\theta_{1}⊗\theta_{2}=(\mu_{1}\mu_{2},\eta_{1}+\eta_{2}-\eta_{1}\eta_{2},v_{1}+v_{2}-v_{1}v_{2})$;
3. $\lambda \theta_{1}=(1-{\left(1-\mu_{1}\right)}^{\lambda },{\left(\eta_{1}\right)}^{\lambda },{\left(v_{1}\right)}^{\lambda }),\lambda >0$;
4. $\theta_{1}^{\lambda }=({\left(\mu_{1}\right)}^{\lambda },1-{\left(1-\eta_{1}\right)}^{\lambda },1-{\left(1-v_{1}\right)}^{\lambda }),\lambda >0$.

### 3.2. Interval-valued picture fuzzy set

Cuong et al. (2013) proposed IVPFS theory based on PFS [24]. This theory allows for a more flexible representation of uncertainty and ambiguity in data, making it highly suitable for decision-making in complex systems [45, 46]. To formalize this concept, let *X* be a given domain of discourse. Let *G*[0,1] be the subintervals set of interval [0,1] and *x*≠0 be the given set. Thus, an IVPFS can be described as follows:

$$A=\{\langle x,\mu_{A}(x),\eta_{A}(x),\nu_{A}(x)\rangle |x\in X\}$$ (3)

Where $\mu_{A}:x\to G[0,1],\eta_{A}:x\to G[0,1],\nu_{A}:x\to G[0,1],\forall x\in X,0\leq sup(\mu_{A}(x)+sup(\eta_{A}(x)+\sup\left(\nu_{A}(x)\right)\leq 1$. The intervals *μ*_*A*_(*x*), *η*_*A*_(*x*), *ν*_*A*_(*x*) represent positive, negative and neutral membership degrees of *A*, and $\mu_{A}^{L}(x),\mu_{A}^{U}(x),\eta_{A}^{L}(x),\eta_{A}^{U}(x),\nu_{A}^{L}(x),\nu_{A}^{U}(x)$ represent the lower and upper end points. Then, an interval-valued picture fuzzy set *A* can be written as:

$$A=\{\langle x,[\mu_{A}^{L}(x),\mu_{A}^{U}(x)],[\eta_{A}^{L}(x),\eta_{A}^{U}(x)],[\nu_{A}^{L}(x),\nu_{A}^{U}(x)]\rangle |x\in X\}$$ (4)

Where $\mu_{A}^{L}(x)\geq 0,\eta_{A}^{L}(x)\geq 0\&\nu_{A}^{L}(x)\geq 0$ and $0\leq \mu_{A}^{U}(x)+\eta_{A}^{U}(x)+\nu_{A}^{U}(x)\leq 1$. Refusal membership degree expressed by *π*_*A*_ can be determined using the formula below (5).


$$\pi_{A}=[\pi_{A}^{L}(x),\pi_{A}^{U}(x)]=[1-(\mu_{A}^{U}(x)+\eta_{A}^{U}(x)+\nu_{A}^{U}(x)],1-(\mu_{A}^{L}(x)+\eta_{A}^{L}(x)+\nu_{A}^{L}(x))]$$ (5)

**Definition 3** For two IVPFNs *A* = (*μ*_*A*_(*x*), *η*_*A*_(*x*), *ν*_*A*_(*x*)) and *B* = (*μ*_*B*_(*x*), *η*_*B*_(*x*), *ν*_*B*_(*x*)). α as a scalar value *α*>0. The following shows the basic and significant operations of IVPFS:

1.
$A⊕B=(\left[\mu_{A}^{L}+\mu_{B}^{L}-\mu_{A}^{L}\mu_{B}^{L},\mu_{A}^{U}+\mu_{B}^{U}-\mu_{A}^{U}\mu_{B}^{U}],[\eta_{A}^{L}\eta_{B}^{L},\eta_{A}^{U}\eta_{B}^{U}],[\nu_{A}^{L}\nu_{B}^{L},\upsilon_{A}^{U}\nu_{B}^{U}\right])$

2.
$A⊗B=(\left[\mu_{A}^{L}\mu_{B}^{L},\mu_{A}^{U}\mu_{B}^{U}],[\eta_{A}^{L}+\eta_{B}^{L}-\eta_{A}^{L}\eta_{B}^{L},\eta_{A}^{U}+\eta_{B}^{U}-\eta_{A}^{U}\eta_{B}^{U}],[\nu_{A}^{L}+\nu_{B}^{L}-\nu_{A}^{L}\nu_{B}^{L},\nu_{A}^{U}+\nu_{B}^{U}-\nu_{A}^{U}\nu_{B}^{U}\right)]$

3.
$A^{\alpha }=(\left[{\left(\mu_{A}^{L}\right)}^{\alpha },{\left(\mu_{A}^{U}\right)}^{\alpha }],[1-{\left(1-\eta_{A}^{L}\right)}^{\alpha },1-{\left(1-\eta_{A}^{U}\right)}^{\alpha }],[1-{\left(1-\nu_{A}^{L}\right)}^{\alpha },1-{\left(1-\nu_{A}^{U}\right)}^{\alpha }\right])$

4.
$\alpha A=(\left[1-{\left(1-\mu_{A}^{L}\right)}^{\alpha },1-{\left(1-\mu_{A}^{U}\right)}^{\alpha }],[{\left(\eta_{A}^{L}\right)}^{\alpha },{\left(\eta_{A}^{U}\right)}^{\alpha }],[{\left(\nu_{A}^{L}\right)}^{\alpha },{\left(\nu_{A}^{U}\right)}^{\alpha }\right])$

The operations based on IVPFS can give the following properties:

Suppose that *M*, *N* & *H* are the three IVPFNs, where M = (μ_M_(x), η_M_(x), ν_M_(x)) and *N* = (*μ*_*N*_(*x*), *η*_*N*_(*x*), *ν*_*N*_(*x*)) and *H* = (*μ*_*H*_(*x*), *η*_*H*_(*x*), *ν*_*H*_(*x*)) and α>0, therefore:

-
M⊕N=N⊕M

-
(M⊕N)⊕H=M⊕(N⊕H)

-
α(M⊕N)=αM⊕αN

-
M⊗N=N⊗M

-
(M⊗N)⊗H=M⊗(N⊗H)

**Definition 4** Let that $A_{i}=([\mu_{i}^{L},\mu_{i}^{U}],\;[\eta_{i}^{L},\eta_{i}^{U}],\;[\nu_{i}^{L},\nu_{i}^{U}])(i=1,2,\cdots ,n)$ be the IVPFN, *Ω* is the set of whole IVPFNs. *ω* = (*ω*_1_, *ω*_2_,⋯,*ω*_*n*_)^*T*^ as the weight vector of them, a mapping IVPFOWIA: *Ω*^*n*^→*Ω* of dimension *n* is an interval-valued picture fuzzy ordered weighted interactive averaging (IVPFOWIA) operator, with $\sum_{i=1}^{n}\omega_{i}=1,\;\omega_{i}=[0,1]$. Then,

$$\mathrm{IVPFOWIA}\left(A_{1},A_{2},\ldots ,A_{n}\right)=\overset{n}{\underset{i=1}{⨁\left(\omega_{i}A_{i}\right)}}$$ (6)

Let $A_{i}=(\left[\mu_{i}^{L},\mu_{i}^{U}],\;[\eta_{i}^{L},\eta_{i}^{U}],\;[\nu_{i}^{L},\nu_{i}^{U}\right])$ be a collection of IVPFNs, then by Eq (6) and the weighted interaction operational laws in Definition 4, the aggregated result by using the IVPFOWIA operator is also an IVPFN, and then:

$$\mathrm{IVPFOWIA}(A_{1},A_{2},\ldots ,A_{n})=\overset{n}{\underset{i=1}{⨁}}(\omega_{i}A_{i})=\langle [1-\prod_{i=1}^{n}{\left(1-\mu_{i}^{L}\right)}^{\omega_{i}},1-\prod_{i=1}^{n}{\left(1-\mu_{i}^{U}\right)}^{\omega_{i}}],\left[\prod_{i=1}^{n}{\left(1-\mu_{i}^{L}\right)}^{\omega_{i}}-\prod_{i=1}^{n}{\left(1-\mu_{i}^{L}-\eta_{i}^{L}\right)}^{\omega_{i}},\prod_{i=1}^{n}{\left(1-\mu_{i}^{U}\right)}^{\omega_{i}}-\prod_{i=1}^{n}{\left(1-\mu_{i}^{U}-\eta_{i}^{U}\right)}^{\omega_{i}}\right],\left[\prod_{i=1}^{n}{\left(1-\mu_{i}^{L}-\eta_{i}^{L}\right)}^{\omega_{i}}-\prod_{i=1}^{n}{\left(1-\mu_{i}^{L}-\eta_{i}^{L}-\nu_{i}^{L}\right)}^{\omega_{i}},\prod_{i=1}^{n}{\left(1-\mu_{i}^{U}-\eta_{i}^{U}\right)}^{\omega_{i}}-\prod_{i=1}^{n}{\left(1-\mu_{i}^{U}-\eta_{i}^{U}-\nu_{i}^{U}\right)}^{\omega_{i}}]\right\rangle$$ (7)

Where *ω* = (*ω*_1_, *ω*_2_,⋯,*ω*_*n*_)^*T*^ is the weight of *A*_*i*_(*i* = 1,2,⋯,*n*), such that $\sum_{i=1}^{n}\omega_{i}=1,\;\omega_{i}=[0,1]$.

**Appendix A** provides the proof for this theorem.

**Definition 5** Let $A_{i}=([\mu_{i}^{L},\mu_{i}^{U}],\;[\eta_{i}^{L},\eta_{i}^{U}],\;[\nu_{i}^{L},\nu_{i}^{U}])$ be an IVPFN, then the score function *S*(*A*_*i*_) and the accuracy function *H*(*N*_*i*_) of the IVPFNs can be described as:

$$\begin{array}{l} S(A_{i})=\frac{\mu_{i}^{L}-\eta_{i}^{L}-\nu_{i}^{L}+\mu_{i}^{U}-\eta_{i}^{U}-\nu_{i}^{U}}{2},\;S(A_{i})\in [-1,1]\\ H(A_{i})=\frac{\mu_{i}^{L}+\eta_{i}^{L}+\nu_{i}^{L}+\mu_{i}^{U}+\eta_{i}^{U}+\nu_{i}^{U}}{2},\;H(A_{i})\in [0,1] \end{array}$$ (8)

In the light of the equation of the score function *S*(*A*_*i*_) and the accuracy function *H*(*A*_*i*_) given in Definition 5, the size of the two IVPFNs can be compared by the following equation:

1. Supposing that *S*(*A*_1_)<*S*(*A*_2_), so that *A*_1_<*A*_2_
2. Supposing that *S*(*A*_1_) = *S*(*A*_2_), so that:
  - Supposing that *H*(*A*_1_)<*H*(*A*_2_) so that *A*_1_<*A*_2_
  - Supposing that *H*(*A*_1_) = <*H*(*A*_2_) so that *A*_1_ = *A*_2_

The rule can be drawn from the above equation: the larger the score function S(A_i_) is, the larger the IVPFN is.

**Definition 6** Let $A_{1}=(\left[\mu_{1}^{L},\mu_{1}^{U}],\;[\eta_{1}^{L},\eta_{1}^{U}],\;[\nu_{1}^{L},\nu_{1}^{U}\right]),\;A_{2}=([\mu_{2}^{L},\mu_{2}^{U}],\;[\eta_{2}^{L},\eta_{2}^{U}],\;[\nu_{2}^{L},\nu_{2}^{U}])$ be two IVPFNs, so the Hamming distance of *A*_1_ and *A*_2_ is as follows:

$$D_{H}(A_{1},A_{2})=\frac{1}{6}\{{|\mu }_{1}^{L}-\mu_{2}^{L}|+{|\mu }_{1}^{U}-\mu_{2}^{U}|+{|\eta }_{1}^{L}-\eta_{2}^{L}|+{|\eta }_{1}^{U}-\eta_{2}^{U}|+{|\nu }_{1}^{L}-\nu_{2}^{L}|+{|\nu }_{1}^{U}-\nu_{2}^{U}|\}$$ (9)

The Euclidean distance of *A*_1_ and *A*_2_ is as follows:

$$D_{E}(A_{1},A_{2})=\frac{1}{6}\sqrt{\left\{{\left({|\mu }_{1}^{L}-\mu_{2}^{L}|+{|\mu }_{1}^{U}-\mu_{2}^{U}|\right)}^{2}+{\left({|\eta }_{1}^{L}-\eta_{2}^{L}|+{|\eta }_{1}^{U}-\eta_{2}^{U}|\right)}^{2}+{\left({|\nu }_{1}^{L}-\nu_{2}^{L}|+{|\nu }_{1}^{U}-\nu_{2}^{U}|\right)}^{2}\right\}}$$ (10)

### 3.3. The entropy of interval-valued picture fuzzy set

#### 3.3.1. Shannon’s entropy

As a prevalent technique for weight determination, Shannon’s entropy objectively assigns weights to indicators based on the amount of information they provide and the correlation between them [2, 47, 48], and it has been used in areas such as product design, environmental protection and medicine and health [48]. In order to mitigate the influence of human-induced discrepancies, Shannon’s entropy is employed to compute weights by quantifying the extent of divergence among individual index values. According to Wang and Lee [49], The specific calculation of Shannon’s entropy is as follows:

Suppose that a team of decision makers has been given the task of weighing *m* alternatives against *n* design criteria. Let *D* = [*y*_*ij*_] be the decision matrix, where 1<*i*<*m* and 1<*j*<*n*.

**Step 1:** Standardize the decision matrix *D*, and eliminate differences in scale between criteria.


$$\tau_{ij}=\frac{y_{ij}}{\sum_{i=1}^{m}y_{ij}}$$ (11)

**Step 2:** Compute the information entropy matrix of criterion *E* = (*e*_*j*_) by Eq (12).Where *e*_*j*_ denotes *j*^th^ criterion’s entropy value.


$$e_{j}=-k\sum_{i=1}^{m}\tau_{ij}\cdot \ln\;\tau_{ij}$$ (12)

In Eq (12): *k* is a constant, we can use formula k=1lnm to calculate it.

**Step 3:** Determine the weight ($w_{j}^{\prime }$) of the criteria element, which is calculated as follows:

$$w_{j}^{\prime }=\frac{1-e_{j}}{\Sigma_{j=1}^{n}(1-e_{j})}$$ (13)

In Eq (13), $\sum_{j=1}^{n}w_{j}^{\prime }=1,\;w_{j}^{\prime }\geq 0$.

#### 3.3.2. The entropy of IVPFS

Due to the properties of the information environment, Shannon entropy has various extensions. For example, Burillo and Bustince [50] introduced entropy of IFS, and Chen [2] employed entropy of rough set (RS) in design concept evaluation. Joshi and Kumar [51] defined the entropy of interval-valued intuitionistic hesitant fuzzy set (IVIHFS), by using the entropy of IVIHFS to calculate the weights of criteria. Ye [52] introduced a fuzzy cross entropy of the interval-valued intuitionistic fuzzy sets (IVIFS) to determine the weights of schemes. Qiyas et al. [53] proposed the entropy of generalized interval-valued picture fuzzy linguistic set (GIVPFLS), which is defined in detail in Definition 7. Based on the literature review on entropy weight mentioned above, we can see that many scholars have provided entropy measures for RS, IFS, IVIHFS, IVIFS, and GIVPFLS. In this manuscript, we utilize the entropy weighting method proposed by Qiyas et al. In MCDM, IVPFS exhibits a notable advantage in addressing decision-maker hesitancy and uncertainty. A key feature of IVPFS is its incorporation of abstention intervals, which proves invaluable in handling decision-maker hesitancy [54]. When decision-makers encounter uncertainty regarding certain elements or attributes, or when they cannot definitively ascertain the membership and non-membership degrees of elements, they can employ abstention intervals to signify their hesitancy. These abstention intervals allow decision-makers to express the extent of their uncertainty within a specified interval range, without the necessity of making explicit decisions. This flexibility is particularly pertinent for complex real-world decision problems where decision-makers regularly confront uncertainty and conflicting information. The entropy of IVPFS is a useful method, especially in circumstances when decision-makers are unable to convey their preferences about criteria during the early phases of the decision-making process or are unsure of how to do so. This approach accounts for the degree of uncertainty pertaining to the alternatives offered by decision-makers and the divergence among various aspects within the IVPFS, permitting the straightforward computation of the relative relevance of the criteria.

**Definition 7** [53] let $\hat{H_{i}}=\langle \left[H_{\alpha },H_{\beta }];[{\ddot{a}}_{\hat{H_{i}}}^{l},{\ddot{a}}_{\hat{H_{i}}}^{u}],[{\ddot{e}}_{\hat{H_{i}}}^{l},{\ddot{e}}_{\hat{H_{i}}}^{u}],\lfloor {\ddot{u}}_{{\hat{H}}_{i}}^{l},{\ddot{u}}_{{\hat{H}}_{i}}^{u}\right\rfloor \rangle$ be a separate element in an IVPFLS $\hat{H}$. For this, entropy is defined as.


$$E(\hat{H_{i}})=\frac{1}{3}\left(1-\frac{\left|{\ddot{a}}_{\hat{H_{i}}}^{l}-{\ddot{e}}_{\hat{H_{i}}}^{l}-{\ddot{u}}_{{\hat{H}}_{i}}^{l}|+|{\ddot{a}}_{\hat{H_{i}}}^{u}-{\ddot{e}}_{\hat{H_{i}}}^{u}-{\ddot{u}}_{{\hat{H}}_{i}}^{u}\right|}{3}\right)\left(1+\frac{\pi_{\hat{H_{i}}}^{l}+\pi_{\hat{H_{i}}}^{u}}{3}\right)\;i=1,2,\cdots ,n.$$

According to the Definition 7, we derive the calculation formula for the entropy method of IVPFS.


$$e_{j}=\frac{1}{m}\sum_{i=1}^{m}\frac{\left(3-|\mu_{A}^{L}(x_{i})-\eta_{A}^{L}(x_{i})-\nu_{A}^{L}(x_{i})|-|\mu_{A}^{U}(x_{i})-\eta_{A}^{U}(x_{i})-\nu_{A}^{U}(x_{i})|)(3+\pi_{A}^{L}(x_{i})+\pi_{A}^{U}(x_{i})\right)}{9}$$ (14)

Subsequently, the subsequent formula is employed for the computation of attribute weights.


$$w_{j}=\frac{1-e_{j}}{\sum_{j=1}^{m}1-e_{j}}$$ (15)

for all *j* = 1,2,⋯,*n*.

## 4. Proposed methodology

**Fig 1:**
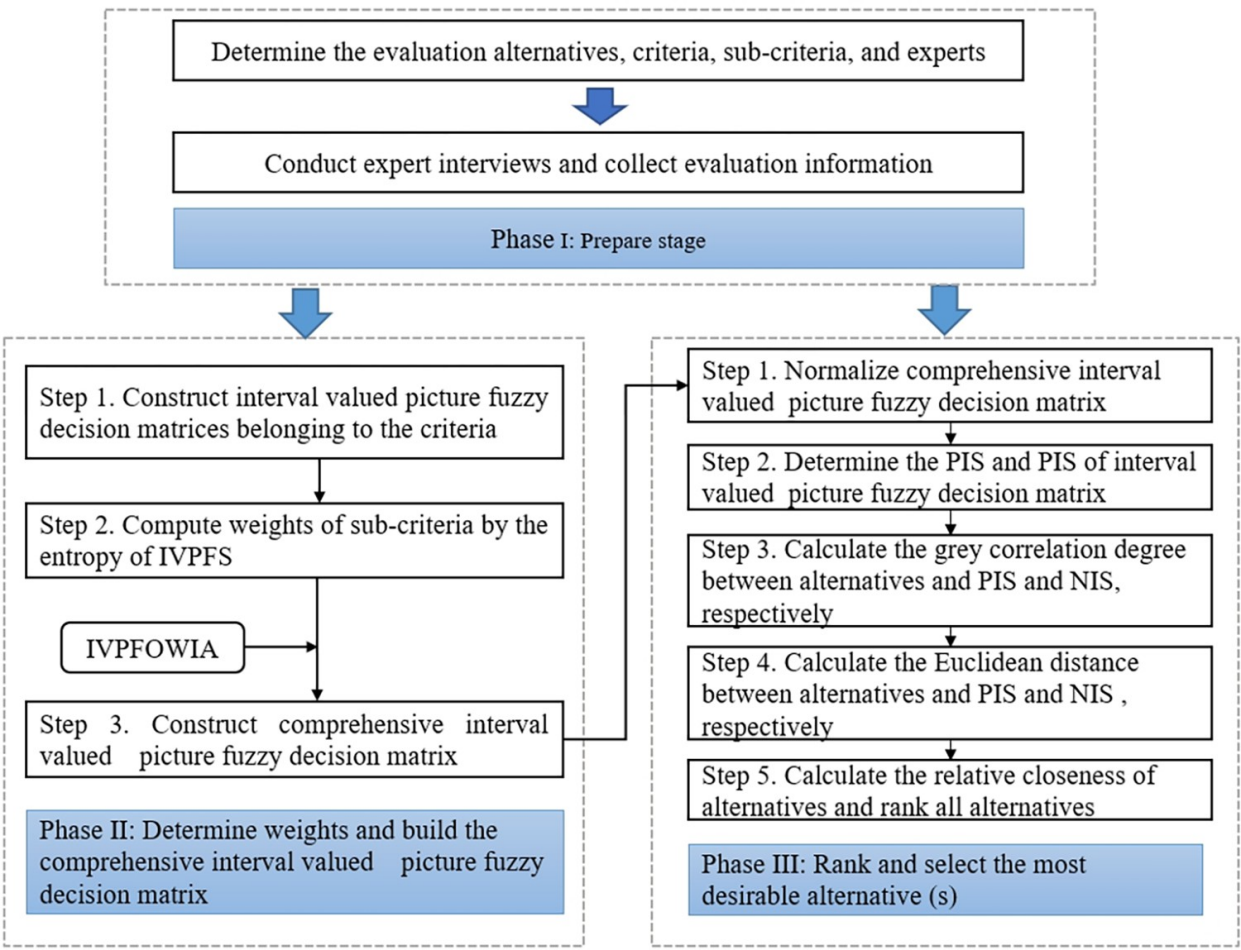
Flowchart of the IVPFS-MCDM for design concept evaluation. Phase I is the preparation stage, which mainly includes expert interviews, literature collection, determination of alternative solutions, and evaluation index system. Phase II mainly includes the determination of criteria weights and the construction of a comprehensive evaluation matrix. Phase III mainly involves determining the optimal design alternative.

In this section we provide a novel framework for choosing leisure yachts design alternatives that is based on IVPFS and the improved TOPSIS technique. The procedure phases of the IVPFS-Improved TOPSIS method are depicted in Fig 1. There are three phases: (1) prepare phase, (2) construct the collective IVPF decision matrix, and (3) sort and select the best design scheme. Phase 1 involves establishing the design concept’s assessment index system by making use of the body of existing research and the expertise of decision makers. In phase 2, the weights of criterion layer as well as indicator layer can be calculated through the entropy of IVPFS. With the help of IVPFOWIA, the collective IVPF decision matrix can be constructed. In phase 3, the TOPSIS technique is improved in the IVPFS to compute the relative closeness of design concept schemes to positive and negative ideal solutions. The final ranking of different design concept solutions can be obtained.

### 4.1 Prepare phase

**Fig 2:**
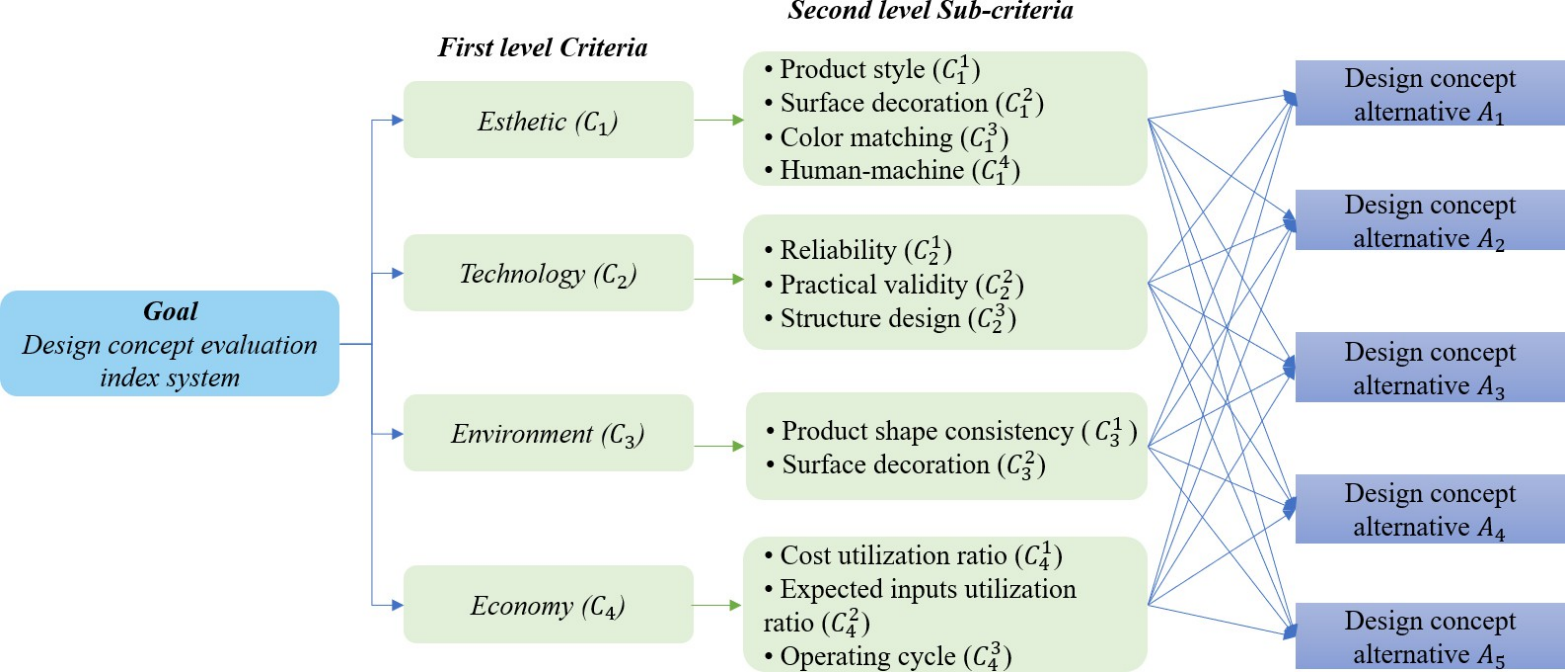
Design concept evaluation index system. The design evaluation index system is mainly divided into goal layer and criteria layer. The criteria layer includes first level criteria and second level sub-criteria layer.

It is vital to determine each of the assessment criteria and sub-criteria during the design concept formulation and selection process [55]. The evaluation indicators for proposing design concepts can be determined from the perspectives of product economic benefits, technology, and environmental protection. The design concepts evaluation index system has been constructed from four aspects: aesthetic evaluation, technical evaluation, economic evaluation, and environmental evaluation. We have collected and summarized literature on design concept evaluation in recent years, and found that certain indicators have appeared multiple times in the design concept evaluation indicator system. In order to identify the most appropriate indicators for the evaluation of design concepts, a panel of decision-makers from relevant fields such as industrial design and design concepts was invited to this study. By communicating with the expert group, analyzing and determining the evaluation indicators of design concept, a design concept evaluation indicator system is constructed, as shown in Fig 2. For each standard layer and its sub-standard layers, literature sources are provided in Table 1.

**Table 1:** Design concept evaluation index system.

Goal	standard layer	sub-standard layer	References
Design concept evaluation index system	Esthetic (*C*_1_)	Product style ($C_{1}^{1}$)	[56, 57]
		Surface decoration ($C_{1}^{2}$)	[58]
		Color matching ($C_{1}^{3}$)	[59]
		Human-machine ($C_{1}^{4}$)	[58]
	Technology (*C*_2_)	Reliability ($C_{2}^{1}$)	[12, 60]
		Practical validity ($C_{2}^{2}$)	Expert interview
		Structure design ($C_{2}^{3}$)	[59]
	Environment (*C*_3_)	Recycling consideration ($C_{3}^{1}$)	[56]
		Green and pollution-free ($C_{3}^{2}$)	[61]
	Economy (*C*_4_)	Cost utilization ratio ($C_{4}^{1}$)	[12] [59 62]
		Expected inputs utilization ratio ($C_{4}^{2}$)	Expert interview
		Operating cycle ($C_{4}^{3}$)	[11, 58]

### 4.2 Determine comprehensive evaluation decision matrix

According to the index system in Table 1, for such decision problems as design concept evaluation, the indicators are portrayed by qualitative methods, which have uncertainty and imprecision. In this regard, there are generally two types of measurement methods: a quantitative measurement method, where each indicator is artificially assigned a deterministic value within a specified range of values; and a qualitative level measurement method, where each indicator is assigned a certain level within a specified range of levels. Therefore, the latter method is often used to avoid extreme evaluation events and reflect the fuzzy characteristics of fuzzy indicators, such as using A, B, C, and D levels. Of course, there are also extreme events, some are assigned A and some are assigned D. However, the dispersion of such ratings is obviously much smaller for different ratings given by multiple decision-makers. If we can adopt a scientific approach to normalize the statistics and apply them scientifically without losing the information contained in them, the latter must be better than the former. To this end, this research proposes a new index measurement method based on the connotation of IVPFNs and its generation mechanism.

For the MCDM problem of design concept evaluation, we assume *D* = {*D*_1_, *D*_2_,⋯,*D*_*k*_} is the set of *k* DMs, *C* = {*C*_1_, *C*_2_,⋯,*C*_*n*_} is the set of *n* design criteria, *A* = {*A*_1_, *A*_2_,…,*A*_*m*_} is the set of *m* design schemes. The weights of design criteria are presented by *w* = (*w*_1_, *w*_2_,⋯,*w*_*j*_), where $\sum_{j=1}^{n}w_{j}=1,\;0\leq w_{j}\leq 1$. The next sections discuss the specifics of the established design concept alternative evaluation model based on these assumptions.

**Step 1:** Construct interval-valued picture fuzzy decision matrices belonging to the criteria.

Based on the views of all decision makers, under the *l*^*th*^ sub-criteria $C_{j}^{l}$ with respect to criterion *C*_*j*_, IVPF evaluation values of alternatives $A_{i}:\;y_{j}^{il}=(\mu_{j}^{il},\eta_{j}^{il},\nu_{j}^{il})\;i=1,2,\cdots ,m\;j=1,2,\cdots ,n\;l=1,2,\cdots n_{j}.$


$$y_{j}^{il}=(\mu_{j}^{il},\eta_{j}^{il},\nu_{j}^{il})=[\mu_{jL}^{il},\mu_{jU}^{il}],\;[\eta_{jL}^{il},\eta_{jU}^{il}],\;[\nu_{jL}^{il},\nu_{jU}^{il}])$$ (16)

**Fig 3:**
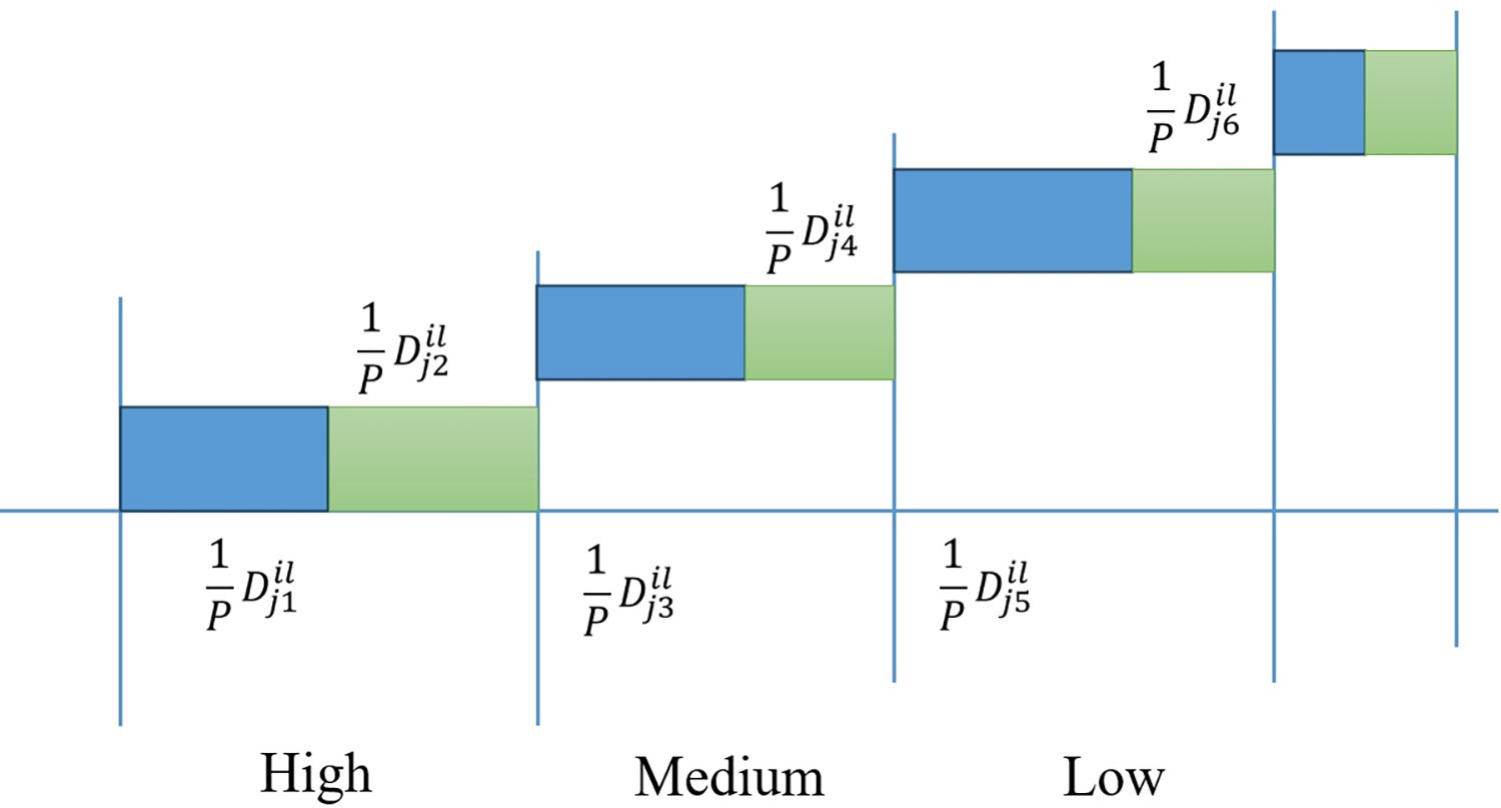
IVPFN composition graph. This figure includes four evaluation options (High、Medium、Low and Difficult to judge). Each evaluation option has two attitudes, namely affirmative and hesitate.

The specific calculation steps are as follows:

First, for sub-criteria $C_{j}^{l}$ with respect to criteria *C*_*j*_, each decision-maker evaluates *n* design concept alternatives. The evaluation opinions are provided by the questionnaire survey in Table 2 and Fig 3.

**Table 2:** Sub-criteria evaluation questionnaire.

Sub-criteria	Evaluation options	Attitude	Statistical results	Proportion
$C_{j}^{l}$	High	Affirmative	$D_{j1}^{il}$	$\frac{1}{P}D_{j1}^{il}$
		Hesitate	$D_{j2}^{il}$	$\frac{1}{P}D_{j2}^{il}$
	Medium	Affirmative	$D_{j3}^{il}$	$\frac{1}{P}D_{j3}^{il}$
		Hesitate	$D_{j4}^{il}$	$\frac{1}{P}D_{j4}^{il}$
	Low	Affirmative	$D_{j5}^{il}$	$\frac{1}{P}D_{j5}^{il}$
		Hesitate	$D_{j6}^{il}$	$\frac{1}{P}D_{j6}^{il}$
	Difficult to judge	Abstention	$D_{j7}^{il}$	$\frac{1}{P}D_{j7}^{il}$

Second, for data statistics, for the second level evaluation indicator $C_{j}^{l}$ with respect to criteria *C*_*j*_, calculate the evaluation opinions of all decision-makers on *n* alternatives. $y_{j}^{il}$ can be calculated by Eq (17).


$$\{\begin{array}{c} \mu_{jL}^{il}=\frac{D_{j1}^{il}}{P},\mu_{jU}^{il}=\frac{D_{j1}^{il}+D_{j2}^{il}}{P}\\ \eta_{jL}^{il}=\frac{D_{j3}^{il}}{P},\eta_{jU}^{il}=\frac{D_{j3}^{il}+D_{j4}^{il}}{P}\\ \nu_{jL}^{il}=\frac{D_{j5}^{il}}{P},\nu_{jU}^{il}=\frac{D_{j5}^{il}{+D}_{j6}^{il}}{P} \end{array},\;j=1,2,\cdots ,n$$ (17)

Where *P* represents the number of all evaluation experts. $D_{j1}^{il}$ denotes the number of evaluation experts who hold a high (affirmative) attitude towards the design concept alternative *A*_*i*_ relative to the sub-criterion $C_{j}^{l}.D_{j2}^{il}$ denotes the number of evaluation experts who hold a high (hesitate) attitude towards the design concept alternative *A*_*i*_ relative to the sub-criterion $C_{j}^{l}.D_{j3}^{il}$ denotes the number of evaluation experts who hold a medium (affirmative) attitude towards the design concept alternative *A*_*i*_ relative to the sub-criterion $C_{j}^{l}.D_{j4}^{il}$ denotes the number of evaluation experts who hold a medium (hesitate) attitude towards the design concept alternative *A*_*i*_ relative to the sub-criterion $C_{j}^{l}.D_{j5}^{il}$ denotes the number of evaluation experts who hold a low (affirmative) attitude towards the design concept alternative *A*_*i*_ relative to the sub-criterion $C_{j}^{l}.D_{j6}^{il}$ denotes the number of evaluation experts who hold a low (hesitate) attitude towards the design concept alternative *A*_*i*_ relative to the sub-criterion $C_{j}^{l}$.

By using Eq (17), building the IVPF evaluation matrix $Y_{j}={\left(y_{j}^{il}\right)}_{m\times n_{j}}$ corresponding to sub-criteria $C_{j}^{l}.(l=1,2,\cdots ,n_{j})$ belonging to the criterion *C*_*j*_.


$$\begin{array}{l} \begin{array}{cccc} \;C_{j}^{1} & C_{j}^{2} & \cdots & C_{j}^{n_{j}} \end{array}\\ Y_{ij}=\begin{array}{cc} \begin{array}{c} A_{1}\\ \vdots \\ A_{i}\\ \vdots \\ A_{m} \end{array} & [\begin{array}{cccc} y_{j}^{11} & y_{j}^{12} & \cdots & y_{j}^{1n_{j}}\\ \vdots & \vdots & & \vdots \\ y_{j}^{i1} & y_{j}^{i2} & \cdots & y_{j}^{in_{j}}\\ \vdots & \vdots & \cdots & \vdots \\ y_{j}^{m1} & y_{j}^{m2} & \cdots & y_{j}^{mn_{j}} \end{array}],j=1,2,\ldots ,n. \end{array} \end{array}$$ (18)

**Step 2:** Construct comprehensive IVPF decision matrix.

the weight of the sub-criteria $C_{l}^{j}$ can be computed using Eqs (14)–(15). Through applying Eq (19), compute the aggregated value ${\tilde{r}}_{ij}=(\left[\mu_{ij}^{L},\mu_{ij}^{U}],\;[\eta_{ij}^{L},\eta_{ij}^{U}],\;[\nu_{ij}^{L},\nu_{ij}^{U}\right])$ for design concept scheme *A*_*i*_ relative to criterion *C*_*j*_ by employing IVPFOWIA.


$${\tilde{r}}_{ij}=\mathrm{IVPFOWIA}(y_{j}^{i1},y_{j}^{i1}\cdots {,y}_{j}^{in_{j}},)=\overset{n}{\underset{i=1}{⨁}}(w_{j}^{l}y_{j}^{il})=\langle [1-\prod_{i=1}^{n}{\left(1-\mu_{jL}^{il}\right)}^{w_{j}^{l}},1-\prod_{i=1}^{n}{\left(1-\mu_{jU}^{il}\right)}^{w_{j}^{l}}],[\prod_{i=1}^{n}{\left(1-\mu_{jL}^{il}\right)}^{w_{j}^{l}}-\prod_{i=1}^{n}{\left(1-\mu_{jL}^{il}-\eta_{jL}^{il}\right)}^{w_{j}^{l}},\prod_{i=1}^{n}{\left(1-\mu_{jL}^{il}\right)}^{w_{j}^{l}}-\prod_{i=1}^{n}{\left(1-\mu_{jU}^{il}-\eta_{jU}^{il}\right)}^{w_{j}^{l}}],\left[\prod_{i=1}^{n}{\left(1-\mu_{jL}^{il}-\eta_{jL}^{il}\right)}^{w_{j}^{l}}-\prod_{i=1}^{n}{\left(1-\mu_{jL}^{il}-\eta_{jL}^{il}-\nu_{jL}^{il}\right)}^{w_{j}^{l}},\prod_{i=1}^{n}{\left(1-\mu_{jU}^{il}-\eta_{jU}^{il}\right)}^{w_{j}^{l}}-\prod_{i=1}^{n}{\left(1-\mu_{jU}^{il}-\eta_{jU}^{il}-\nu_{jU}^{il}\right)}^{w_{j}^{l}}]\right\rangle =(\left[\mu_{ij}^{L},\mu_{ij}^{U}],[\eta_{ij}^{L},\eta_{ij}^{U}],[\nu_{ij}^{L},\nu_{ij}^{U}\right])$$ (19)

Finally, the comprehensive IVPF decision matrix $\tilde{R}={\left({\tilde{r}}_{ij}\right)}_{m\times n}$ can be determined by using the IVPFOWIA operator given in Eq (20):

$$\tilde{R}={\left({\tilde{r}}_{ij}\right)}_{m\times n}=[\begin{array}{cccc} {\tilde{r}}_{11} & {\tilde{r}}_{12} & \cdots & {\tilde{r}}_{1n}\\ {\tilde{r}}_{21} & {\tilde{r}}_{22} & \cdots & {\tilde{r}}_{2n}\\ \vdots & \vdots & \vdots & \vdots \\ {\tilde{r}}_{m1} & {\tilde{r}}_{m2} & \cdots & {\tilde{r}}_{mn} \end{array}]$$ (20)

### 4.3 Determine and choose the best design concept alternative(s)

In this phase, we employ the improved TOPSIS technique based on IVPFS to rank the best design concept scheme. Following are the detailed steps:

**Step 1:** Standardize the decision-making assessment matrix. To counteract the impact of varying criteria scales on the decision-making procedure, it is essential to render the initial matrix dimensionless.Therefore, in this step, we transform the initialized judgement matrix $\tilde{R}={\left({\tilde{r}}_{ij}\right)}_{m\times n}$ into a normalized decision matrix ${\tilde{R}}^{*}={\left({\tilde{r}}_{ij}^{*}\right)}_{m\times n}$.


$${\tilde{r}}_{ij}^{*}=\{\begin{array}{l} r_{ij},\;if\;C_{j}\;is\;a\;benefit‐type\;criterion,\\ {\overset{-}{r}}_{ij},\;if\;C_{j}\;is\;a\;cost‐type\;criterion. \end{array}$$ (21)

Where *i* = 1,2,⋯*m*, *j* = 1,2⋯,*n*.

**Step 2:** Utilizing the standardized judgment matrix, ascertain the IVPF-positive ideal solution (*R*^+^) and the IVPF-negative ideal solution (*R*^−^):

$$\left[R^{+}]=[{\tilde{r}}_{1}^{+},{\tilde{r}}_{2}^{+},{\tilde{r}}_{3}^{+},\cdots ,{\tilde{r}}_{n}^{+}\right]$$ (22)

$$\left[R^{-}]=[{\tilde{r}}_{1}^{-},{\tilde{r}}_{2}^{-},{\tilde{r}}_{3}^{-},\cdots ,{\tilde{r}}_{n}^{-}\right]$$ (23)

Where:

$${\tilde{r}}_{j}^{+}=\{\begin{array}{c} \mu_{j}^{L+}=\underset{i}{max}\mu_{ij}^{L}\\ \mu_{j}^{U+}=\underset{i}{max}\mu_{ij}^{U}\\ \eta_{j}^{L+}=\underset{i}{min}\eta_{ij}^{L}\\ \eta_{j}^{U+}=\underset{i}{min}\eta_{ij}^{U}\\ \nu_{j}^{L+}=\underset{i}{min}\nu_{ij}^{L}\\ \nu_{j}^{U+}=\underset{i}{min}\nu_{ij}^{U} \end{array}$$ (24)

$${\tilde{r}}_{j}^{-}=\{\begin{array}{c} \mu_{j}^{L-}=\underset{i}{min}\mu_{ij}^{L}\\ \mu_{j}^{U-}=\underset{i}{min}\mu_{ij}^{U}\\ \eta_{j}^{L-}=\underset{i}{min}\eta_{ij}^{L}\\ \eta_{j}^{U-}=\underset{i}{min}\eta_{ij}^{U}\\ \nu_{j}^{L-}=\underset{i}{max}\nu_{ij}^{L}\\ \nu_{j}^{U-}=\underset{i}{max}\nu_{ij}^{U} \end{array}$$ (25)

**Step 3:** Compute the grey correlation degree between alternatives and positive (negative) ideal points, respectively.

i. Compute the grey correlation coefficients $S_{ij}^{+}$ and $S_{ij}^{-}$ between alternatives and the IVPF-PIS (NIS) ${\tilde{r}}_{j}^{+(-)}$, respectively:

$$S_{ij}^{+(-)}=\frac{{min}_{i}{min}_{j}d({\tilde{r}}_{ij},{\tilde{r}}_{j}^{+(-)})+\rho {max}_{i}{max}_{j}d({\tilde{r}}_{ij},{\tilde{r}}_{j}^{+(-)})}{d({\tilde{r}}_{ij},{\tilde{r}}_{j}^{+(-)})+\rho {max}_{i}{max}_{j}d({\tilde{r}}_{ij},{\tilde{r}}_{j}^{+(-)})}$$ (26)

Where *ρ* is called the resolution coefficient. When *ρ* = 0, there is no surrounding environment. Likewise, when *ρ* = 1, nothing changes in the surrounding environment. Usually, $\rho =0.5.d({\tilde{r}}_{ij},{\tilde{r}}_{j}^{+(-)})$ is the distance between ${\tilde{r}}_{ij}$ and ${\tilde{r}}_{j}^{+}({\tilde{r}}_{j}^{-})$ which can be obtained by Eq (9).

ii. Compute the grey correlation degree $\gamma_{i}^{+}$ and $\gamma_{i}^{-}$ between alternatives and IVPF-PIS (NIS).

$$\gamma_{i}^{+}=\sum_{j=1}^{n}w_{j}S_{ij}^{+}$$ (27)

$$\gamma_{i}^{-}=\sum_{j=1}^{n}w_{j}S_{ij}^{-}$$ (28)

**Step 4:** Compute the Euclidean distance between alternatives and IVPF-PIS (NIS).


$$\begin{array}{c} d_{i}^{+}=\sqrt{\sum_{j=1}^{n}(r_{ij}-r_{j}^{+}{)}^{2}}(i=1,2,\cdots ,m,j=1,2,\cdots ,n)\\ d_{i}^{-}=\sqrt{\sum_{j=1}^{n}(r_{ij}-r_{j}^{-}{)}^{2}}(i=1,2,\cdots ,m,j=1,2,\cdots ,n) \end{array}$$ (29)

Normalize $\gamma_{i}^{+},\gamma_{i}^{-},d_{i}^{+}$ and $d_{i}^{-}$ separately, and merge the normalized $\gamma_{i}^{+},\gamma_{i}^{-},d_{i}^{+}$, and $d_{i}^{-}$ combinations to obtain the closeness of $B_{i}^{+}$ and $B_{i}^{-}$ between alternatives and the IVPF-PIS (NIS) $r_{j}^{+(-)}$.


$$\begin{array}{c} B_{i}^{+}=\frac{1}{2}(\frac{\gamma_{i}^{+}}{\max{\gamma_{i}}^{+}}+\frac{d_{i}^{+}}{\max d_{i}^{+}})\\ B_{i}^{-}=\frac{1}{2}(\frac{\gamma_{i}^{-}}{\max\gamma_{i}^{-}}+\frac{d_{i}^{-}}{\max d_{i}^{-}}) \end{array}$$ (30)

**Step 5:** Sort and select the best design concept scheme.

Compute the relative closeness *D*_*i*_ of design concept schemes:

$$D_{i}=\frac{B_{i}^{+}}{{B_{i}^{+}+B}_{i}^{-}}\;i=1,2,\cdots ,m.$$ (31)

The design concept alternatives are ranked and preferred according to their relative closeness *D*_*i*_, with higher values of *D*_*i*_ indicating higher design effectiveness of the alternative. Conversely, the lower the value.

## 5. Empirical example

In this study, the improved TOPSIS method of IVPFS proposed in section 4 is used to select the optimal design scheme from multiple leisure yachts. In this research work, we needed to redevelop a leisure yacht for a yachting company in Weihai, China. All decision-makers are longstanding experts in the field of leisure yachts design, and have extensive design experience. The decision-makers form an evaluation and selection group. There are 30 decision-makers *D* = {*D*_1_, *D*_2_,…,*D*_30_}, and five design concept alternatives *A* = {*A*_1_, *A*_2_, *A*_3_, *A*_4_, *A*_5_}. According to the main procedures of the proposed model, the specific example steps of design concept evaluation are as follows.

### 5.1. Construct the collective IVPF decision matrix

According to the design concept evaluation index system in Table 1, taking leisure yachts design *A*_1_ as an example. This study invited 30 experts to participate in the evaluation. There are 7 experts who have a high (affirmative) attitude towards the leisure yachts alternative *A*_1_ relative to the sub-criterion $C_{2}^{1}$, 5 experts hold a high (hesitate) attitude towards the leisure yachts alternative *A*_1_ relative to to the sub-criterion $C_{2}^{1}$, 5 experts hold a medium (affirmative) attitude towards the leisure yachts alternative *A*_1_ relative to the sub-criterion $C_{2}^{1}$, 3 experts hold a medium (hesitate) attitude towards the leisure yachts alternative *A*_1_ relative to the sub-criterion $C_{2}^{1}$, 4 experts hold a low (affirmative) attitude towards the leisure yachts alternative *A*_1_ relative to the sub-criterion $C_{2}^{1}$, 5 experts hold a low (hesitate) attitude towards the leisure yachts alternative *A*_1_ relative to the sub-criterion $C_{2}^{1}$, and then, taking *P* = 30 in Eq (17), IVPF evaluation value ${y_{2}^{11}=(\mu_{2}^{11},\eta_{2}^{11},\nu }_{2}^{11})=[\mu_{2L}^{11},\mu_{2U}^{11}],[\eta_{2L}^{11},\eta_{2U}^{11}],[\nu_{2L}^{11},\nu_{2U}^{11}])$ can be calculated for leisure yachts alternative A_1_ relative to sub-criteria $C_{2}^{1}$:

$$\left\{ \begin{array}{c} \mu_{2L}^{11}=\frac{1}{P}D_{21}^{11}=\frac{7}{30}=0.23,\mu_{2U}^{11}=\frac{1}{P}D_{21}^{11}+\frac{1}{P}D_{22}^{11}=\frac{7}{30}+\frac{5}{30}=0.40\\ \eta_{2L}^{11}=\frac{1}{P}D_{23}^{11}=\frac{5}{30}=0.17,\eta_{2U}^{11}=\frac{1}{P}D_{23}^{11}+\frac{1}{P}D_{24}^{11}=\frac{5}{30}+\frac{3}{30}=0.27\\ \nu_{2L}^{11}=\frac{1}{P}D_{25}^{11}=\frac{4}{30}=0.13,\nu_{2U}^{11}=\frac{1}{P}D_{25}^{11}+\frac{1}{P}D_{26}^{11}=\frac{4}{30}+\frac{5}{30}=0.30 \end{array}\right.$$

Therefore $y_{2}^{11}=([0.23,0.40],[0.17,0.27],[0.13,0.30])$. In this study, we can also calculate the leisure yachts alternative *A*_1_ relative to the sub-criteria $C_{2}^{2},C_{2}^{3}$ relative to criterion C_2_. The above calculation process is based on A_1_ as an example And then, the IVPF evaluation value for the leisure yachts alternatives *A*_2_−*A*_5_ relative to the sub-criteria $C_{2}^{1},C_{2}^{2}\;and\;C_{2}^{3}$, which are shown in Table 3, is determined using the same calculation procedure. Finally, we can also compute the IVPF evaluation matrix relative to the sub-criteria belonging to criteria (*C*_1_, *C*_3_, *C*_4_) using the same procedure.

**Table 3:** Judge matrices belonging to the sub-criteria of Technology (C2).

	$C_{2}^{1}$	$C_{2}^{2}$	$C_{2}^{3}$
*A*_1_	([.23,.40],[.17,.27],[.13,.30])	([.33,.43],[.13,.27],[.13,.23])	([.33,.43],[.13,.23],[.13,.33])
*A*_2_	([.30,.47],[.10,.30],[.10,.20])	([.20,.33],[.16,.30],[.16,.30])	([.13,.27],[.10,.27],[.13,.30])
*A*_3_	([.33,.50],[.13,.23],[.07,.27])	([.30,.47],[.10,.17],[.07,.20])	([.33,.50],[.10,.17],[.10,.17])
*A*_4_	([.33,.43],[.13,.23],[.10,.27])	([.30,.40],[.17,.30],[.13,.30])	([.27,.37],[.13,.23],[.10,.27])
*A*_5_	([.20,.30],[.13,.30],[.10,.30])	([.30,.47],[.13,.30],[.10,.20])	([.37,.50],[.13,.23],[.13,.23])

*Where: taking A_1_-C_2_ as an example, ([.23,.40],[.17,.27],[.13,.30]) is the abbreviation of ([0.23,0.40],[0.17,0.27],[0.13,0.30]).

The entropy of IVPFS can be used to computed the local weights of sub-criteria, as shown by Eqs (14)–(15):

$$w_{C_{1}}={\left(0.245,0.245,0.260,0.250\right)}^{T}$$

$$w_{C_{2}}={\left(0.313,0.347,0.340\right)}^{T}$$

$$w_{C_{3}}={\left(0.467,0.533\right)}^{T}$$

$$w_{C_{4}}={\left(0.331,0.323,0.36\right)}^{T}$$

By applying the IVPFOWIA, we can obtain the collective decision matrix as shown in Table 4.

**Table 4:** The comprehensive IVPF decision matrix.

	*C*_1_	*C*_2_	*C*_3_	*C*_4_
*A*_1_	([.33,.51],[.08,.21],[.08,.27])	([.30,.42],[.14,.25],[.13,.32])	([.25,.38],[.11,.30],[.15,.31])	([.26,.37],[.14,.27],[.15,.34])
*A*_2_	([.29,.43],[.13,.31],[.11,.27])	([.21,.36],[.12,.29],[.13,.27])	([.30,.45],[.12,.28],[.13,.21])	([.30,.43],[.08,.16],[.09,.22])
*A*_3_	([.26,.45],[.12,.21],[.14,.30])	([.32,.48],[.11,.19],[.07,.18])	([.28,.40],[.13,.28],[.13,.28])	([.23,.34],[.13,.26],[.12,.30])
*A*_4_	([.28,.41],[.13,.33],[.14,.27])	([.28,.40],[.14,.26],[.11,.34])	([.35,.51],[.10,.19],[.08,.20])	([.26,.40],[.13,.21],[.15,.28])
*A*_5_	([.24,.35],[.11,.23],[.20,.41])	([.28,.42],[.13,.28],[.11,.24])	([.30,.46],[.13,.20],[.14,.28])	([.19,.30],[.10,.20],[.19,.38])

With the help of Eqs (14)–(15), we can determine the entropy weights of IVPFS of *C* = {*C*_1_, *C*_2_, *C*_3_, *C*_4_} is *w* = (0.245,0.244,0.251,0.260)^*T*^.

### 5.2. Determine and choose the best design concept alternative(s)

**Step 1:** Due to the four criteria are all benefits (not costs), according to Eq (21), therefore the normalized evaluation decision matrix is consistent with Table 5.

**Table 5:** The standardized IVPF decision matrix.

	*C*_1_	*C*_2_	*C*_3_	*C*_4_
*A*_1_	([.33,.51],[.08,.21],[.08,.27])	([.30,.42],[.14,.25],[.13,.32])	([.25,.38],[.11,.30],[.15,.31])	([.26,.37],[.14,.27],[.15,.34])
*A*_2_	([.29,.43],[.13,.31],[.11,.27])	([.21,.36],[.12,.29],[.13,.27])	([.30,.45],[.12,.28],[.13,.21])	([.30,.43],[.08,.16],[.09,.22])
*A*_3_	([.26,.45],[.12,.21],[.14,.30])	([.32,.48],[.11,.19],[.07,.18])	([.28,.40],[.13,.28],[.13,.28])	([.23,.34],[.13,.26],[.12,.30])
*A*_4_	([.28,.41],[.13,.33],[.14,.27])	([.28,.40],[.14,.26],[.11,.34])	([.35,.51],[.10,.19],[.08,.20])	([.26,.40],[.13,.21],[.15,.28])
*A*_5_	([.24,.35],[.11,.23],[.20,.41])	([.28,.42],[.13,.28],[.11,.24])	([.30,.46],[.13,.20],[.14,.28])	([.19,.30],[.10,.20],[.19,.38])

**Step 2:** The IVPF-PIS and IVPF-NIS of the standardized decision matrix are calculated through Eqs (22)–(23).


$$R^{+}=\{(\left[0.33,0.51],[0.08,0.21],[0.08,0.27\right]),(\left[0.32,0.48],[0.11,0.19],[0.07,0.18\right]),(\left[0.35,0.51],[0.10,0.19],[0.18,0.20\right])(\left[0.26,0.40],[0.13,0.21],[0.15,0.28\right])\}$$

$$R^{-}=\{(\left[0.24,0.35],[0.11,0.23],[0.20,0.41\right]),(\left[0.21,0.36],[0.12,0.29],[0.13,0.27\right]),(\left[0.25,0.38],[0.11,0.30],[0.15,0.31\right])(\left[0.19,0.30],[0.10,0.20],[0.19,0.38\right])\}$$

**Step 3:** Determine the grey correlation degree between alternatives and positive (negative) ideal points, respectively.

Determine the grey correlation coefficients $S_{ij}^{+}$ and $S_{ij}^{-}$ of each alternative with the positive (negative) ideal point sets $r_{j}^{+}$ and $r_{j}^{-}$ by using Eq (26), and then calculate the grey correlation degree $\gamma_{i}^{+}$ and $\gamma_{i}^{-}$ of each alternative with the positive (negative) ideal point sets $r_{j}^{+}$ and $r_{j}^{-}$ based on Eqs (27)–(28). The results obtained are shown in Table 6.

**Table 6:** Grey correlation degree $\gamma_{i}^{+}\;and\;\gamma_{i}^{-}$.

Grey correlation degree	Design concept evaluation alternatives				
	*A*_1_	*A*_2_	*A*_3_	*A*_4_	*A*_5_
$\gamma_{i}^{+}$	0.576	0.451	0.619	0.725	0.435
$\gamma_{i}^{-}$	0.587	0.551	0.486	0.421	0.754

**Step 4:** Compute the Euclidean distance between alternatives and IVPF-PIS (NIS) by Eq (29). The results obtained are shown in Table 7.

**Table 7:** Euclidean distance.

Euclidean distance	Design concept evaluation alternatives				
	*A*_1_	*A*_2_	*A*_3_	*A*_4_	*A*_5_
$d_{i}^{+}$	0.177	0.226	0.147	0.133	0.192
$d_{i}^{-}$	0.275	0.243	0.293	0.200	0.082

**Step 5:** Select the best design concept scheme.

Compute the relative closeness *D*_*i*_ of each design concept alternative through Eqs (30)–(31). The specific parameters and alternatives are listed in Table 8.

**Table 8:** The order of the five design concept alternatives.

	$B_{i}^{+}$	$B_{i}^{-}$	IVPF-Improved TOPSIS method	
			*D*_*i*_	Rank
A_1_	0.788	0.858	0.521	2
A_2_	0.811	0.780	0.490	3
A_3_	0.752	0.822	0.522	1
A_4_	0.794	0.620	0.439	5
A_5_	0.725	0.640	0.469	4

According to the values of *D*_*i*_, it can be concluded that the ranking order is *A*_3_>*A*_1_>*A*_2_>*A*_5_>*A*_4_.

### 5.3. Sensitivity analysis

**Fig 4:**
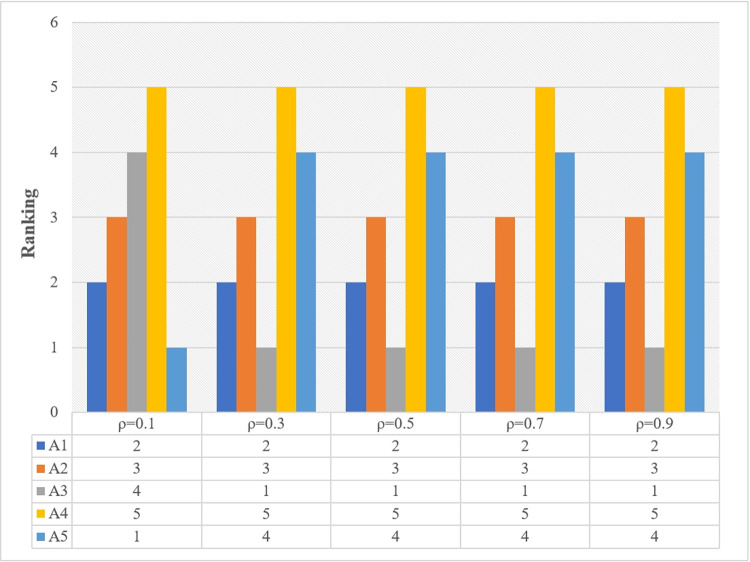
Results of sensitivity analysis. This figure presents the sensitivity analysis results for IVPF-improved TOPSIS method at different *ρ* values (ρ = 0.1, 0.3, 0.5, 0.7, 0.9). When *ρ* = 0.1, due to *ρ* The value of is relatively small, which may make the model less sensitive to changes in parameters. Therefore, even slight changes in parameters may result in different sorting results. This is because the smaller *ρ* The value reduces the sensitivity threshold to changes in weights and evaluation values.

In this section, we conduct the sensitivity analysis of the resolution coefficient *ρ* in the proposed IVPF-Improved TOPSIS method. When *ρ* = 0.5, the ranking of the five design concept solutions is *A*_3_>*A*_1_>*A*_2_>*A*_5_>*A*_4_. Fig 4 shows the results of the ranking of the alternatives for different values of the resolution coefficients *ρ*. According to Fig 4, it can be seen that in addition to *ρ* = Beyond 0.1, *A*_3_ is always the best of the five design concept alternatives. Sensitivity analysis shows the stability and reliability of the model proposed in this study.

### 5.4. Comparative analysis

**Fig 5:**
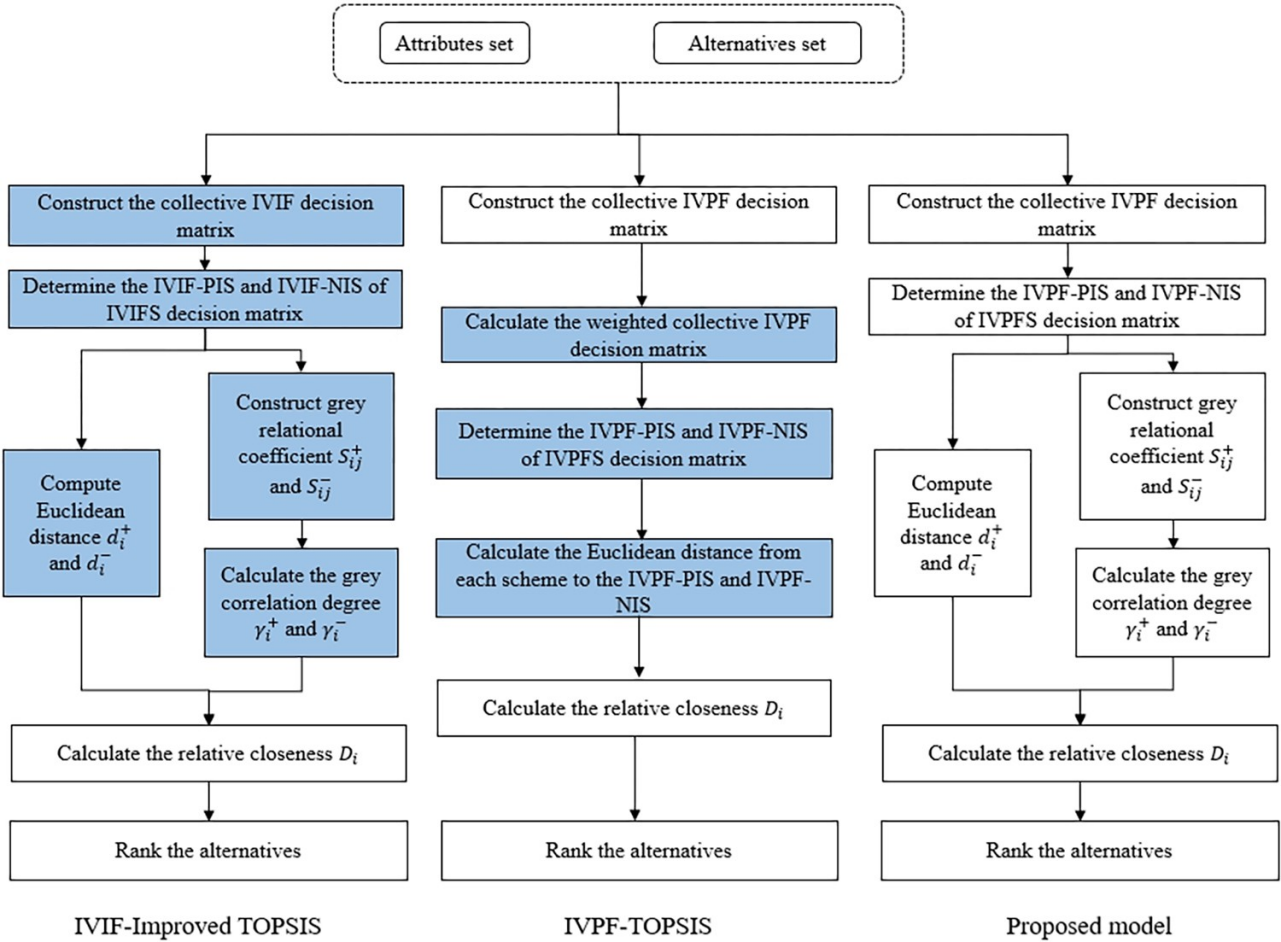
Comparison of different models. This figure illustrates the comparison between various models and their performance in the evaluation process.

**Fig 6:**
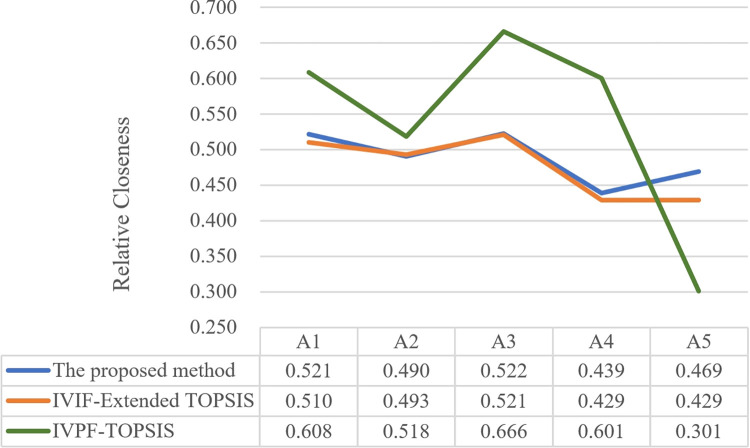
Relative closeness of different MCDM methods. This figure depicts the relative closeness and comparative analysis of various methods used in the study.

In order to evaluate and verify the effectiveness of the proposed algorithm in this study, comparison studies are done in addition to the case study using interval-valued intuitionistic fuzzy (IVIF)-Extended TOPSIS method and IVPF-TOPSIS method. Fig 5 compares several models, while Table 9 and Fig 6 present the findings of a comparison of various methodologies.

**Table 9:** The results of comparisons between different methods.

Alternatives	IVIF-Extended TOPSIS		IVPF-TOPSIS		Proposed method	
	*D*_*i*_	Rank	*D*_*i*_	Rank	*D*_*i*_	Rank
A_1_	0.512	2	0.609	2	0.521	2
A_2_	0.493	3	0.518	4	0.490	3
A_3_	0.521	1	0.665	1	0.523	1
A_4_	0.429	4	0.600	3	0.438	5
A_5_	0.428	5	0.301	5	0.469	4

From Fig 6, we can see that the proposed model and the other two models obtain the same optimal solution. The differences are reflected in the entire process of design optimization or some data processing steps. The details are as follows:

1. IVIF improved TOPSIS method. The difference between this method and our model is that the fuzzy environment is different. The IVPFS proposed in this research considers IVIFS at the same time. In addition, it also takes into account the neutrality of expert evaluation information, which is closer to the real evaluation process.
2. IVPF-TOPSIS method. The difference between this and our proposed method is that this research improves the TOPSIS method. The improved TOPSIS model solves the problem of large data fluctuations by calculating the degree of correlation between evaluation objects. From Table 9, it can also be seen that the difference in relative closeness between the five design alternatives obtained by the method proposed in this research is smaller, and the difference in relative closeness values between the five design alternatives obtained using IVPF-TOPSIS is significant. Thus, the method proposed in this manuscript has strong sensitivity.

**Fig 7:**
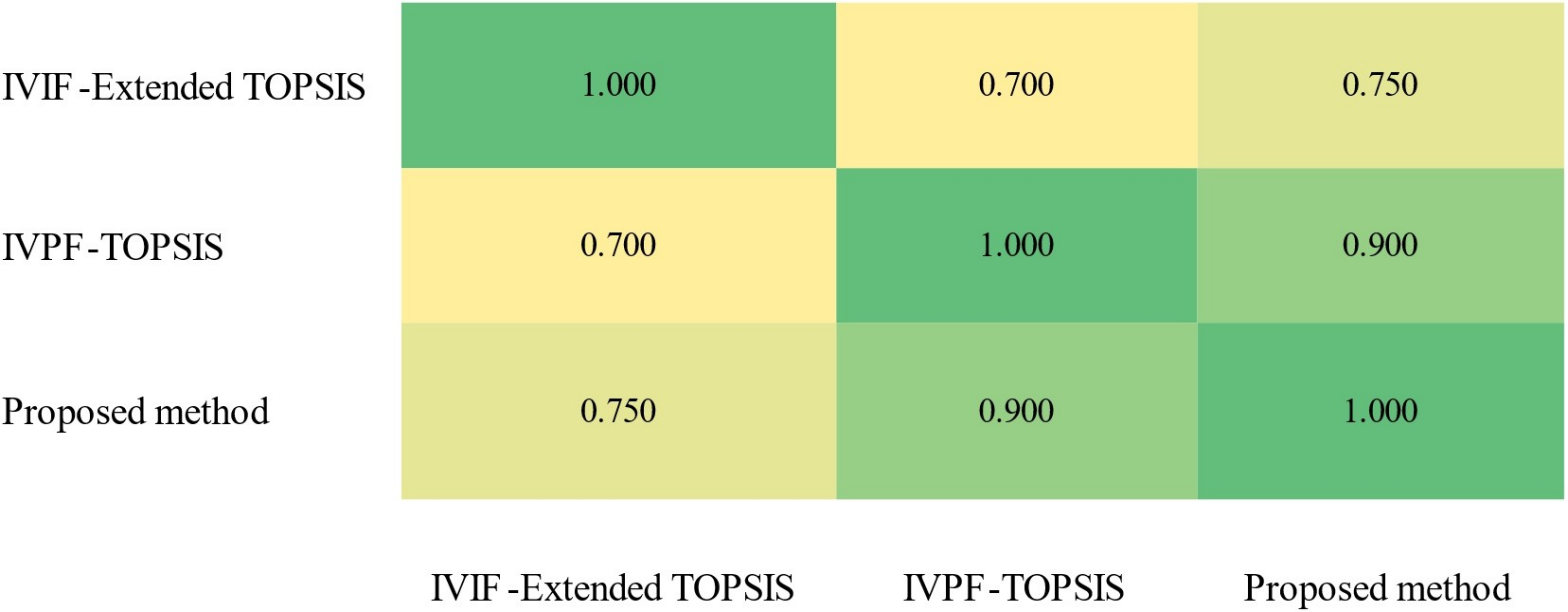
Spearman correlation coefficients between the results of the different methods. This figure presents the Spearman correlation coefficients that measure the relationships between the results obtained from various evaluation methods.

The Spearman correlation coefficients among the results of the three methods are shown in Fig 7. It can be clearly seen from the Fig 7 that the correlation between the IVIF-Extended TOPSIS and IVPF-TOPSIS methods is not very high (Spearman correlation coefficient is less than 0.8), because the IVIF-Extended TOPSIS method did not consider neutrality, and the IVPF-TOPSIS method did not consider the shortcomings of TOPSIS method in its evaluation. The Fig 7 also shows that the correlation between the IVPF-TOPSIS method and the proposed IVPF-improved TOPSIS is very high, up to 0.900. However, the sorting order between these two methods is not exactly the same, indicating the effectiveness of improved TOPSIS method based on IVPFS.

## 6. Conclusion

Design concept evaluation is an essential step in the product development process. The objective of this manuscript is to introduce an innovative approach for evaluation design concepts that takes into account multiple criteria while addressing the challenges of uncertainty and imprecise information. The main contributions of this manuscript can be summarized as follows:

- This study effectively addressed fuzziness and uncertainty in the design concept evaluation using IVPFS theory. Specifically, this work provided a practical method to convert qualitative assessment data into IVPFNs for adaptable decision-making in design evaluation.
- This study considered the advantages of the OWIA operator and combines IVPFS and OWIA operators to form the IVPFOWIA operator, which is used to aggregate expert decision information into a comprehensive decision matrix.
- This study combined the improved TOPSIS method with IVPFS theory to evaluate various design concepts and determine the best one. This approach overcomes the limitation of traditional TOPSIS, which could not account for dynamic changes in the evaluation index sequence, thus ensuring the accuracy of decision outcomes.

The results of the alternatives ranking and the comparative analysis validate the accuracy and scientific validity of the IVPFS-improved TOPSIS model presented in this study. As shown in this manuscript, the provided design concept evaluation and choose algorithms can also be used to deal with other uncertain decision-making problems.

In this manuscript, there are still some limitations that need to be addressed in subsequent research: (i) The methods of objectively calculating weights often overlook important information related to subjective factors, which may result in decision results not being sufficient to reflect the actual situation, as they cannot capture the influence of human subjective experience and knowledge. (ii) In this study, improved TOPSIS method cannot eliminate the nonlinear correlation between indexes.

In the future research, we will analyze and build design concept evaluation index system from perspective of producers, designers and users. we will also explore other operators to combine with IVPFS, enabling the model to adapt to different decision-making scenarios and requirements. In addition, we intend to improve more decision making method, such as ANP and MABAC methods, in order to combine with IVPFS for ranking alternatives.

### Appendix A

**Proof.** By applying mathematical induction to positive integer *n*, we demonstrate that Eq (7) holds.

When *n* = 1, we have:

$$\omega_{1}A_{1}=\langle [1-{\left(1-\mu_{1}^{L}\right)}^{\omega_{1}},1-{\left(1-\mu_{1}^{U}\right)}^{\omega_{1}}],[{\left(1-\mu_{1}^{L}\right)}^{\omega_{1}}-{\left(1-\mu_{1}^{L}-\eta_{1}^{L}\right)}^{\omega_{1}},{\left(1-\mu_{1}^{U}\right)}^{\omega_{1}}-{\left(1-\mu_{1}^{U}-\eta_{1}^{U}\right)}^{\omega_{1}}],\left[{\left(1-\mu_{1}^{L}-\eta_{1}^{L}\right)}^{\omega_{1}}-{\left(1-\mu_{1}^{L}-\eta_{1}^{L}-\nu_{1}^{L}\right)}^{\omega_{1}},{\left(1-\mu_{1}^{U}-\eta_{1}^{U}\right)}^{\omega_{1}}-{\left(1-\mu_{1}^{U}-\eta_{1}^{U}-\nu_{1}^{U}\right)}^{\omega_{1}}]\right\rangle$$

Thus, Eq (7) holds for *n* = 1.

Assuming that the Eq (7) also holds when *n* = *k*, then:

$$\mathrm{IVPFOWIA}(A_{1},A_{2},\ldots ,A_{k})=\overset{k}{\underset{i=1}{⨁}}(\omega_{i}A_{i})=\langle [1-\prod_{i=1}^{k}{\left(1-\mu_{i}^{L}\right)}^{\omega_{i}},1-\prod_{i=1}^{k}{\left(1-\mu_{i}^{U}\right)}^{\omega_{i}}],\left[\prod_{i=1}^{k}{\left(1-\mu_{i}^{L}\right)}^{\omega_{i}}-\prod_{i=1}^{k}{\left(1-\mu_{i}^{L}-\eta_{i}^{L}\right)}^{\omega_{i}},\prod_{i=1}^{k}{\left(1-\mu_{i}^{U}\right)}^{\omega_{i}}-\prod_{i=1}^{k}{\left(1-\mu_{i}^{U}-\eta_{i}^{U}\right)}^{\omega_{i}}\right],\left[\prod_{i=1}^{k}{\left(1-\mu_{i}^{L}-\eta_{i}^{L}\right)}^{\omega_{i}}-\prod_{i=1}^{k}{\left(1-\mu_{i}^{L}-\eta_{i}^{L}-\nu_{i}^{L}\right)}^{\omega_{i}},\prod_{i=1}^{k}{\left(1-\mu_{i}^{U}-\eta_{i}^{U}\right)}^{\omega_{i}}-\prod_{i=1}^{k}{\left(1-\mu_{i}^{U}-\eta_{i}^{U}-\nu_{i}^{U}\right)}^{\omega_{i}}]\right\rangle$$

Then, when *n* = *k*+1, by inductive assumption and Definition 4, we have

$$\overset{k+1}{\underset{i=1}{⨁}}(\omega_{i}A_{i})=\overset{k}{\underset{i=1}{⨁}}(\omega_{i}A_{i})⊕(\omega_{k+1}A_{k+1})=\langle [1-\prod_{i=1}^{k}{\left(1-\mu_{i}^{L}\right)}^{\omega_{i}},1-\prod_{i=1}^{k}{\left(1-\mu_{i}^{U}\right)}^{\omega_{i}}],\left[\prod_{i=1}^{k}{\left(1-\mu_{i}^{L}\right)}^{\omega_{i}}-\prod_{i=1}^{k}{\left(1-\mu_{i}^{L}-\eta_{i}^{L}\right)}^{\omega_{i}},\prod_{i=1}^{k}{\left(1-\mu_{i}^{U}\right)}^{\omega_{i}}-\prod_{i=1}^{k}{\left(1-\mu_{i}^{U}-\eta_{i}^{U}\right)}^{\omega_{i}}\right],\left[\prod_{i=1}^{k}{\left(1-\mu_{i}^{L}-\eta_{i}^{L}\right)}^{\omega_{i}}-\prod_{i=1}^{k}{\left(1-\mu_{i}^{L}-\eta_{i}^{L}-\nu_{i}^{L}\right)}^{\omega_{i}},\prod_{i=1}^{k}{\left(1-\mu_{i}^{U}-\eta_{i}^{U}\right)}^{\omega_{i}}-\prod_{i=1}^{k}{\left(1-\mu_{i}^{U}-\eta_{i}^{U}-\nu_{i}^{U}\right)}^{\omega_{i}}]\right\rangle ⊕\langle [1-{\left(1-\mu_{k+1}^{L}\right)}^{\omega_{k+1}},1-{\left(1-\mu_{k+1}^{U}\right)}^{\omega_{k+1}}],[{\left(1-\mu_{k+1}^{L}\right)}^{\omega_{k+1}}-{\left(1-\mu_{k+1}^{L}-\eta_{k+1}^{L}\right)}^{\omega_{k+1}},{\left(1-\mu_{k+1}^{U}\right)}^{\omega_{k+1}}-{\left(1-\mu_{k+1}^{U}-\eta_{k+1}^{U}\right)}^{\omega_{k+1}}],\left[{\left(1-\mu_{k+1}^{L}-\eta_{k+1}^{L}\right)}^{\omega_{k+1}}-{\left(1-\mu_{k+1}^{L}-\eta_{k+1}^{L}-\nu_{k+1}^{L}\right)}^{\omega_{k+1}},{\left(1-\mu_{k+1}^{U}-\eta_{k+1}^{U}\right)}^{\omega_{k+1}}-{\left(1-\mu_{k+1}^{U}-\eta_{k+1}^{U}-\nu_{k+1}^{U}\right)}^{\omega_{k+1}}]\right\rangle =\langle [1-\prod_{i=1}^{k+1}{\left(1-\mu_{i}^{L}\right)}^{\omega_{i}},1-\prod_{i=1}^{k+1}{\left(1-\mu_{i}^{U}\right)}^{\omega_{i}}],\left[\prod_{i=1}^{k+1}{\left(1-\mu_{i}^{L}\right)}^{\omega_{i}}-\prod_{i=1}^{k+1}{\left(1-\mu_{i}^{L}-\eta_{i}^{L}\right)}^{\omega_{i}},\prod_{i=1}^{k+1}{\left(1-\mu_{i}^{U}\right)}^{\omega_{i}}-\prod_{i=1}^{k+1}{\left(1-\mu_{i}^{U}-\eta_{i}^{U}\right)}^{\omega_{i}}\right],\left[\prod_{i=1}^{k+1}{\left(1-\mu_{i}^{L}-\eta_{i}^{L}\right)}^{\omega_{i}}-\prod_{i=1}^{k+1}{\left(1-\mu_{i}^{L}-\eta_{i}^{L}-\nu_{i}^{L}\right)}^{\omega_{i}},\prod_{i=1}^{k+1}{\left(1-\mu_{i}^{U}-\eta_{i}^{U}\right)}^{\omega_{i}}-\prod_{i=1}^{k+1}{\left(1-\mu_{i}^{U}-\eta_{i}^{U}-\nu_{i}^{U}\right)}^{\omega_{i}}]\right\rangle$$

$$=IVPFOWIA(A,A_{2},\ldots ,A_{k+1})$$

The conclusion that Eq (7) is true for positive integer *n* = *k*+1 follows from this. After that, it is true for all positive integers *n*.

## Supporting information

S1 Data (DOCX)

## References

[1] Qi J, Hu J, Peng Y. Modified rough VIKOR based design concept evaluation method compatible with objective design and subjective preference factors. Applied Soft Computing. 2021;107. doi: 10.1016/j.asoc.2021.107414 WOS:000663737200006.

[2] Chen Z, Zhong P, Liu M, Sun H, Shang K. A novel hybrid approach for product concept evaluation based on rough numbers, shannon entropy and TOPSIS-PSI. Journal of Intelligent & Fuzzy Systems 2021;40:12087–99. doi: 10.3233/JIFS-210184 WOS:000667508800118.

[3] Bozorg-Haddad O, Zolghadr-Asli B, Loaiciga HA. A handbook on multi-attribute decision-making methods: John Wiley & Sons; 2021.

[4] Shidpour H, Da Cunha C, Bernard A. Group multi-criteria design concept evaluation using combined rough set theory and fuzzy set theory. Expert Systems with Applications. 2016;64:633–44. doi: 10.1016/j.eswa.2016.08.022 WOS:000383810800050.

[5] Qi J, Hu J, Huang H, Peng Y. New customer-oriented design concept evaluation by using improved Z-number-based multi-criteria decision-making method. Advanced Engineering Informatics. 2022;53. doi: 10.1016/j.aei.2022.101683 WOS:000825367100003.

[6] Chan L-K, Wu M-L. A systematic approach to quality function deployment with a full illustrative example. Omega. 2005;33(2):119–39.

[7] Zhai L-Y, Khoo L-P, Zhong Z-W. Design concept evaluation in product development using rough sets and grey relation analysis. Expert Systems with Applications. 2009;36(3):7072–9. doi: 10.1016/j.eswa.2008.08.068 WOS:000263817100161.

[8] Zhu G-N, Hu J, Ren H. A fuzzy rough number-based AHP-TOPSIS for design concept evaluation under uncertain environments. Applied Soft Computing. 2020;91. doi: 10.1016/j.asoc.2020.106228 WOS:000535477500013.

[9] Chen Z, Zhong P, Liu M, Ma Q, Si G. An integrated expert weight determination method for design concept evaluation. Scientific Reports. 2022;12(1):6358. doi: 10.1038/s41598-022-10333-6 35428829 PMC9012764

[10] Tiwari V, Jain PK, Tandon P. Product design concept evaluation using rough sets and VIKOR method. Advanced Engineering Informatics. 2016;30(1):16–25. doi: 10.1016/j.aei.2015.11.005 WOS:000370094400002.

[11] Song W, Ming X, Wu Z. An integrated rough number-based approach to design concept evaluation under subjective environments. Journal of Engineering Design. 2013;24(5):320–41.

[12] Zhang JM, Wei XP, Wang J, Ieee, editors. Evaluating design concepts by ranking fuzzy numbers. International Conference on Machine Learning and Cybernetics; 2003 2003 Nov 02–05; Xian, PEOPLES R CHINA2003.

[13] Carnahan J, Thurston D, Liu T. Fuzzing ratings for multiattribute design decision-making. Journal of Mechanical Design. 1994.

[14] Akay D, Kulak O, editors. Evaluation of product design concepts using grey-fuzzy information axiom. IEEE International Conference on Grey Systems and Intelligent Services; 2007 2007 Nov 18–20; Nanjing, PEOPLES R CHINA2007.

[15] Geng X, Chu X, Zhang Z. A new integrated design concept evaluation approach based on vague sets. Expert Systems with Applications. 2010;37(9):6629–38. doi: 10.1016/j.eswa.2010.03.058 WOS:000278424600059.

[16] Xu Z, Zhao N. Information fusion for intuitionistic fuzzy decision making: an overview. Information Fusion. 2016;28:10–23.

[17] Hayat K, Ali MI, Alcantud JCR, Cao B-Y, Tariq KU. Best concept selection in design process: An application of generalized intuitionistic fuzzy soft sets. Journal of Intelligent & Fuzzy Systems. 2018;35(5):5707–20. doi: 10.3233/jifs-172121 WOS:000451343900073.

[18] Wang C-H. An intuitionistic fuzzy set–based hybrid approach to the innovative design evaluation mode for green products. Advances in Mechanical Engineering. 2016;8(4):1687814016642715.

[19] Haktanir E, Kahraman C. A novel picture fuzzy CRITIC & REGIME methodology: Wearable health technology application. Engineering Applications Of Artificial Intelligence. 2022;113. doi: 10.1016/j.engappai.2022.104942 WOS:000830168800011.

[20] Zadeh LA. Fuzzy sets. Information & Control. 1965;8(3):338–53. doi: 10.1016/S0019-9958(65)90241-X

[21] Hajiaghaei-Keshteli M, Cenk Z, Erdebilli B, Özdemir YS, Gholian-Jouybari F. PYTHAGOREAN FUZZY TOPSIS METHOD FOR GREEN SUPPLIER SELECTION IN THE FOOD INDUSTRY. Expert Systems with Applications. 2023;224. doi: 10.1016/j.eswa.2023.120036 WOS:000982581000001.

[22] El-Morsy S. Stock Portfolio Optimization Using Pythagorean Fuzzy Numbers. Journal of Operational and Strategic Analytics. 2023;1(1):8–13. doi: 10.56578/josa010102

[23] Atanassov KT. Intuitionistic fuzzy sets. Fuzzy Sets & Systems. 1986;20(1):87–96.

[24] Cuong BC, Kreinovich V, Ieee, editors. Picture Fuzzy Sets—a new concept for computational intelligence problems. Third World Congress on Information and Communication Technologies (WICT); 2013 2013 Dec 15–18; Hanoi, VIETNAM2013.

[25] Cao G, Shen LX. A novel parameter similarity measure between interval-valued picture fuzzy sets with its application in pattern recognition. 2023;- 44(6):- 10239.

[26] Kahraman C, Onar SC, Oztaysi B. Cloud Service Provider Selection Using Interval-Valued Picture Fuzzy TOPSIS. 2022; 504: 507.

[27] Kahraman C, Oztaysi B, Onar S. A Novel Interval Valued Picture Fuzzy TOPSIS Method: Application on Supplier Selection. 2022; 39 (5–6): 635.

[28] Wei G. Picture fuzzy aggregation operators and their application to multiple attribute decision making. Journal of Intelligent & Fuzzy Systems 2017;33(2):713–24. doi: 10.3233/jifs-161798 WOS:000406149900004.

[29] Saha A, Reddy J, Kumar R. A Fuzzy Similarity Based Classification with Archimedean-Dombi Aggregation Operator. Journal of Intelligent Management Decision. 2022;1(2):118–27. doi: 10.56578/jimd010205

[30] Senapati T, Chen GY, Mesiar R, Yager RR. Intuitionistic fuzzy geometric aggregation operators in the framework of Aczel-Alsina triangular norms and their application to multiple attribute decision making. Expert Systems with Applications. 2023;212. doi: 10.1016/j.eswa.2022.118832 WOS:000870058400003.

[31] Liu P, Chen SM. Group Decision Making Based on Heronian Aggregation Operators of Intuitionistic Fuzzy Numbers. IEEE Transactions on Cybernetics. 2017;47(9):2514–30. doi: 10.1109/TCYB.2016.2634599 28029636

[32] Zeng SZ, Hu YJ, Llopis-Albert C. Stakeholder-inclusive multi-criteria development of smart cities. Journal Of Business Research. 2023;154. doi: 10.1016/j.jbusres.2022.08.045 WOS:000863222800001.

[33] Batool B, Abdullah S, Ashraf S, Ahmad M. Pythagorean probabilistic hesitant fuzzy aggregation operators and their application in decision-making. Kybernetes. 2022;51(4):1626–52. doi: 10.1108/k-11-2020-0747 WOS:000661923000001.

[34] Luo MX, Long HF. Picture Fuzzy Geometric Aggregation Operators Based on a Trapezoidal Fuzzy Number and Its Application. Symmetry-Basel. 2021;13(1). doi: 10.3390/sym13010119 WOS:000611837900001.

[35] Garg H. Some Picture Fuzzy Aggregation Operators and Their Applications to Multicriteria Decision-Making. Arabian Journal for Science and Engineering. 2017;42(12):5275–90. doi: 10.1007/s13369-017-2625-9 WOS:000414788400022.

[36] Zoghi M, Rostami G, Khoshand A, Motalleb F. Material selection in design for deconstruction using Kano model, fuzzy-AHP and TOPSIS methodology. Waste Management and Research. 2022;40(4):410–9. doi: 10.1177/0734242X211013904 33928814

[37] Baydas M, Elma OE, Pamucar D. Exploring the specific capacity of different multi criteria decision making approaches under uncertainty using data from financial markets. Expert Systems with Applications. 2022;197. doi: 10.1016/j.eswa.2022.116755 WOS:000792298400004.

[38] Jiang F, Ma L, Broyd T, Chen WY, Luo HB. Digital twin enabled sustainable urban road planning. Sustainable Cities And Society. 2022;78. doi: 10.1016/j.scs.2021.103645 WOS:000780336800002.

[39] Jin CX, Mi JS, Li FC, Liang MS. A novel probabilistic hesitant fuzzy rough set based multi-criteria decision-making method. Information Sciences. 2022;608:489–516. doi: 10.1016/j.ins.2022.06.085 WOS:000864028900006.

[40] Wang YB, Jia XL, Zhang LX. Evaluation of the survival of Yangtze finless porpoise under probabilistic hesitant fuzzy environment. International Journal Of Intelligent Systems. 2022;37(10):7665–84. doi: 10.1002/int.22898 WOS:000788628800001.

[41] Hwang CL, Yoon K, Hwang CL, Yoon K. Multiple Attribute Decision Making. Lecture Notes in Economics & Mathematical Systems. 1981;404(4):287–8.

[42] Saaty TL. What is the Analytic Hierarchy Process? Mathematical Modelling. 1988;48:3–13. doi: 10.1007/978-3-642-83555-1_5

[43] Ju Y, Ju D, Santibanez Gonzalez EDR, Giannakis M, Wang A. Study of site selection of electric vehicle charging station based on extended GRP method under picture fuzzy environment. Computers and Industrial Engineering. 2019;135:1271–85. doi: 10.1016/j.cie.2018.07.048 WOS:000482244100094.

[44] Sivaloganathan AMKaS. Development of a methodology for using function analysis in flexible design strategies. doi: 10.1243/0954405981515635

[45] Masmali I, Hassan R, Shuaib U, Razaq A, Razzaque A, Alhamzi G. Stock Reordering Decision Making under Interval Valued Picture Fuzzy Knowledge. 2023; 15 (4).

[46] Gocer F. A Novel Interval Value Extension of Picture Fuzzy Sets Into Group Decision Making: An Approach to Support Supply Chain Sustainability in Catastrophic Disruptions. Ieee Access. 2021;9:117080–96. doi: 10.1109/access.2021.3105734 WOS:000690419100001.

[47] Shumaiza, Akram M, Al-Kenani AN, Alcantud JCR. Group Decision-Making Based on the VIKOR Method with Trapezoidal Bipolar Fuzzy Information. Symmetry. 2019;11(10):1313. doi: 10.3390/sym11101313

[48] Tiwari V, Jain PK, Tandon P. An integrated Shannon entropy and TOPSIS for product design concept evaluation based on bijective soft set. Journal of Intelligent Manufacturing. 2017.

[49] Wang T-C, Lee H-D. Developing a fuzzy TOPSIS approach based on subjective weights and objective weights. Expert Systems with Applications. 2009;36(5):8980–5. doi: 10.1016/j.eswa.2008.11.035

[50] Burillo P, Bustince H. Entropy on intuitionistic fuzzy sets and on interval-valued fuzzy sets. Fuzzy sets and systems. 1996;78(3):305–16.

[51] Joshi DK, Kumar S. Entropy of interval-valued intuitionistic hesitant fuzzy set and its application to group decision making problems. Granular Computing. 2018.

[52] Ye J. Fuzzy cross entropy of interval-valued intuitionistic fuzzy sets and its optimal decision-making method based on the weights of alternatives. Expert Systems with Applications. 2011;38(5):6179–83. doi: 10.1016/j.eswa.2010.11.052 WOS:000287419900171.

[53] Qiyas M, Abdullah S, Al-Otaibi YD, Aslam M. Generalized interval-valued picture fuzzy linguistic induced hybrid operator and TOPSIS method for linguistic group decision-making. Soft Computing. 2021;25(7):5037–54. doi: 10.1007/s00500-020-05508-0 WOS:000619703000002.

[54] Mahmood T, Waqas HM, Ali Z, Ullah K, Pamucar D. Frank aggregation operators and analytic hierarchy process based on interval-valued picture fuzzy sets and their applications. International Journal Of Intelligent Systems. 2021;36(12):7925–62. doi: 10.1002/int.22614 WOS:000693320300001.

[55] Sun HY, Ma Q, Chen Z, Si GY. A Novel Decision-Making Approach for Product Design Evaluation Using Improved TOPSIS and GRP Method Under Picture Fuzzy Set. International Journal Of Fuzzy Systems. 2023;25(4):1689–706. doi: 10.1007/s40815-023-01471-8 WOS:000936440200001.

[56] Wenyan S, Zixuan N, Pai Z. Design concept evaluation of smart product-service systems considering sustainability: An integrated method. Computers & Industrial Engineering. 2021;159.

[57] Turan FM, Badrul O. The Integration of HOQ and Fuzzy-AHP for Design Concept Evaluation. Applied Mechanics and Materials. 2013;315(1):25–9.

[58] Yang XA, Deng Q, Sun GL, Wang BB. Research on Evaluation and Decision-Making Model of Product Design Scheme Based on TFN-AHP-GRA. Advanced Materials Research. 2011;201–203:1170–6.

[59] Yu SH, DengKai FangWang, editors. Study of information axiom in evaluation method of product design scheme. I Desi & Con Design; 2008.

[60] Kim KJ, Lim CH, Heo JY, Lee DH, Park K. An Evaluation Scheme for Product-Service System Models with a Lifecycle Consideration from Customer’s Perspective. Springer Singapore. 2013.

[61] Kocak SA, Alptekin GI, Bener AB, editors. Evaluation of Software Product Quality Attributes and Environmental Attributes using ANP Decision Framework. International Workshop on Requirements Engineering for Sustainable Systems; 2014.

[62] Wang D, Yu H, Wu J, Meng Q, Lin Q. Integrating fuzzy based QFD and AHP for the design and implementation of a hand training device. J Intell Fuzzy Syst. 2019;36(4):3317–31.

