# Supplementary material for: A novel MCDM approach for design concept evaluation based on interval-valued picture fuzzy sets
Source: PLoS One. 2023 Nov 27;18(11):e0294596. doi: 10.1371/journal.pone.0294596 (PMC10681270; doi:10.1371/journal.pone.0294596)
Supplement: S1 Data — (DOCX) [file pone.0294596.s001.docx]

Raw data

|  |  | | | $A_{1}$ | $A_{2}$ | $A_{3}$ | $A_{4}$ | $A_{5}$ |
| --- | --- | --- | --- | --- | --- | --- | --- | --- |
| $C_{1}$ | $C_{1}^{1}$ | High | Affirmative | 10 | 9 | 6 | 7 | 6 |
|  |  |  | Hesitate | 5 | 4 | 8 | 5 | 5 |
|  |  | Medium | Affirmative | 2 | 4 | 2 | 4 | 4 |
|  |  |  | Hesitate | 3 | 6 | 3 | 5 | 4 |
|  |  | Low | Affirmative | 3 | 3 | 5 | 5 | 6 |
|  |  |  | Hesitate | 3 | 3 | 4 | 4 | 5 |
|  | $C_{1}^{2}$ | High | Affirmative | 12 | 7 | 9 | 8 | 8 |
|  |  |  | Hesitate | 6 | 6 | 4 | 4 | 3 |
|  |  | Medium | Affirmative | 2 | 3 | 2 | 5 | 3 |
|  |  |  | Hesitate | 3 | 5 | 4 | 5 | 4 |
|  |  | Low | Affirmative | 3 | 4 | 4 | 4 | 5 |
|  |  |  | Hesitate | 4 | 4 | 6 | 5 | 6 |
|  | $C_{1}^{3}$ | High | Affirmative | 11 | 9 | 10 | 8 | 7 |
|  |  |  | Hesitate | 5 | 5 | 5 | 4 | 4 |
|  |  | Medium | Affirmative | 3 | 5 | 4 | 4 | 4 |
|  |  |  | Hesitate | 4 | 4 | 2 | 6 | 3 |
|  |  | Low | Affirmative | 2 | 4 | 4 | 4 | 6 |
|  |  |  | Hesitate | 3 | 3 | 4 | 4 | 4 |
|  | $C_{1}^{4}$ | High | Affirmative | 9 | 9 | 7 | 10 | 8 |
|  |  |  | Hesitate | 5 | 3 | 5 | 3 | 2 |
|  |  | Medium | Affirmative | 3 | 4 | 5 | 4 | 3 |
|  |  |  | Hesitate | 4 | 5 | 3 | 6 | 3 |
|  |  | Low | Affirmative | 2 | 3 | 4 | 4 | 6 |
|  |  |  | Hesitate | 3 | 5 | 5 | 2 | 5 |
| $C_{2}$ | $C_{2}^{1}$ | High | Affirmative | 7 | 9 | 10 | 9 | 6 |
|  |  |  | Hesitate | 5 | 5 | 5 | 4 | 3 |
|  |  | Medium | Affirmative | 5 | 3 | 4 | 4 | 4 |
|  |  |  | Hesitate | 3 | 6 | 3 | 3 | 5 |
|  |  | Low | Affirmative | 4 | 3 | 2 | 3 | 3 |
|  |  |  | Hesitate | 5 | 3 | 3 | 5 | 6 |
|  | $C_{2}^{2}$ | High | Affirmative | 10 | 6 | 9 | 9 | 9 |
|  |  |  | Hesitate | 3 | 4 | 5 | 3 | 5 |
|  |  | Medium | Affirmative | 4 | 5 | 3 | 5 | 4 |
|  |  |  | Hesitate | 4 | 4 | 2 | 4 | 5 |
|  |  | Low | Affirmative | 4 | 5 | 2 | 4 | 3 |
|  |  |  | Hesitate | 3 | 4 | 4 | 5 | 3 |
|  | $C_{2}^{3}$ | High | Affirmative | 10 | 4 | 10 | 8 | 11 |
|  |  |  | Hesitate | 3 | 4 | 5 | 3 | 4 |
|  |  | Medium | Affirmative | 4 | 3 | 3 | 4 | 4 |
|  |  |  | Hesitate | 3 | 5 | 2 | 3 | 3 |
|  |  | Low | Affirmative | 4 | 4 | 3 | 3 | 4 |
|  |  |  | Hesitate | 6 | 5 | 2 | 5 | 3 |
| $C_{3}$ | $C_{3}^{1}$ | High | Affirmative | 7 | 8 | 9 | 11 | 9 |
|  |  |  | Hesitate | 4 | 5 | 3 | 5 | 5 |
|  |  | Medium | Affirmative | 4 | 3 | 4 | 3 | 4 |
|  |  |  | Hesitate | 5 | 5 | 5 | 4 | 2 |
|  |  | Low | Affirmative | 4 | 4 | 3 | 2 | 5 |
|  |  |  | Hesitate | 6 | 3 | 5 | 3 | 4 |
|  | $C_{3}^{2}$ | High | Affirmative | 8 | 10 | 8 | 10 | 9 |
|  |  |  | Hesitate | 4 | 4 | 4 | 5 | 5 |
|  |  | Medium | Affirmative | 3 | 4 | 4 | 3 | 4 |
|  |  |  | Hesitate | 6 | 5 | 4 | 2 | 2 |
|  |  | Low | Affirmative | 5 | 4 | 5 | 3 | 4 |
|  |  |  | Hesitate | 1 | 2 | 4 | 4 | 4 |
| $C_{4}$ | $C_{4}^{1}$ | High | Affirmative | 9 | 11 | 9 | 6 | 7 |
|  |  |  | Hesitate | 3 | 5 | 3 | 8 | 3 |
|  |  | Medium | Affirmative | 4 | 3 | 4 | 2 | 3 |
|  |  |  | Hesitate | 3 | 2 | 5 | 3 | 4 |
|  |  | Low | Affirmative | 6 | 2 | 3 | 5 | 5 |
|  |  |  | Hesitate | 3 | 3 | 5 | 4 | 6 |
|  | $C_{4}^{2}$ | High | Affirmative | 8 | 8 | 7 | 8 | 6 |
|  |  |  | Hesitate | 3 | 4 | 3 | 3 | 4 |
|  |  | Medium | Affirmative | 4 | 2 | 3 | 5 | 3 |
|  |  |  | Hesitate | 5 | 3 | 5 | 2 | 2 |
|  |  | Low | Affirmative | 4 | 3 | 4 | 4 | 5 |
|  |  |  | Hesitate | 6 | 4 | 6 | 5 | 7 |
|  | $C_{4}^{3}$ | High | Affirmative | 7 | 9 | 6 | 9 | 5 |
|  |  |  | Hesitate | 4 | 3 | 3 | 3 | 3 |
|  |  | Medium | Affirmative | 4 | 3 | 5 | 4 | 3 |
|  |  |  | Hesitate | 3 | 2 | 2 | 3 | 4 |
|  |  | Low | Affirmative | 4 | 3 | 4 | 5 | 7 |
|  |  |  | Hesitate | 6 | 5 | 3 | 3 | 4 |
